# Cell-Free DNA Fragmentation Patterns in a Cancer Cell Line

**DOI:** 10.3390/diagnostics12081896

**Published:** 2022-08-04

**Authors:** Vida Ungerer, Abel J. Bronkhorst, Carsten Uhlig, Stefan Holdenrieder

**Affiliations:** Munich Biomarker Research Center, Institute of Laboratory Medicine, German Heart Center, Technical University Munich, 80636 Munich, Germany

**Keywords:** cell-free DNA, cfDNA, fragmentomics, cancer, oncology, electrophoresis, liquid biopsy

## Abstract

Unique bits of genetic, biological and pathological information occur in differently sized cell-free DNA (cfDNA) populations. This is a significant discovery, but much of the phenomenon remains to be explored. We investigated cfDNA fragmentation patterns in cultured human bone cancer (143B) cells using increasingly sensitive electrophoresis assays, including four automated microfluidic capillary electrophoresis assays from Agilent, i.e., DNA 1000, High Sensitivity DNA, dsDNA 915 and dsDNA 930, and an optimized manual agarose gel electrophoresis protocol. This comparison showed that (i) as the sensitivity and resolution of the sizing methods increase incrementally, additional nucleosomal multiples are revealed (hepta-nucleosomes were detectable with manual agarose gel electrophoresis), while the estimated size range of high molecular weight (HMW) cfDNA fragments narrow correspondingly; (ii) the cfDNA laddering pattern extends well beyond the 1–3 nucleosomal multiples detected by commonly used methods; and (iii) the modal size of HMW cfDNA populations is exaggerated due to the limited resolving power of electrophoresis, and instead consists of several poly-nucleosomal subpopulations that continue the series of DNA laddering. Furthermore, the most sensitive automated assay used in this study (Agilent dsDNA 930) revealed an exponential decay in the relative contribution of increasingly longer cfDNA populations. This power-law distribution suggests the involvement of a stochastic inter-nucleosomal DNA cleavage process, wherein shorter populations accumulate rapidly as they are fed by the degradation of all larger populations. This may explain why similar size profiles have historically been reported for cfDNA populations originating from different processes, such as apoptosis, necrosis, accidental cell lysis and purported active release. These results not only demonstrate the diversity of size profiles generated by different methods, but also highlight the importance of caution when drawing conclusions on the mechanisms that generate different cfDNA size populations, especially when only a single method is used for sizing.

## 1. Introduction

In the context of cell-free DNA (cfDNA) research, the structural complexity and versatility of the DNA molecule allows at least two unique biological phenomena to occur: first, diverse information can be encoded into the primary structure of DNA as it exists in the nucleus (e.g., organismal and cellular identity); second, as the primary structure of DNA is disrupted and then altered by a variety of processes following its movement from nuclei to intracellular space and various extracellular compartments, it not only conserves the genetic and epigenetic information of the original molecule, but sequences from specific genomic regions may be additionally marked by various physico-chemical features that are often unique to and inform about the characteristics and localization of the processes that generate them (e.g., the type and location of nuclease digestion). As such, a major portion of cfDNA molecules bear a variety of organism, disease as well as tissue-specific genetic and epigenetic physico-chemical features [1,2,3,4].

By virtue of its constant release and short half-life, the capture and computational reconstruction of the information stored in the total cfDNA population or specific cfDNA subpopulations may give an unprecedented, almost real-time view of genome function from which the biological, physiological and pathological state of individuals may be inferred.

The focal point of cfDNA research remains set on the perusal of DNA mutations for the development of routine clinical assays, but at the same time, the field is rapidly expanding towards epigenetic profiling [1,3,4,5]. Given the high frequency and widespread incidence of epigenetic modifications vs. DNA mutations across the genome, it is increasingly appreciated that the profiling of the information encoded into the epigenetic features of cfDNA could significantly expand the window of access for the minimally invasive characterization of genome function and error. On one hand, this will enable the discovery of new and more disease-specific biomarkers and synergize mutational analysis to enhance the analytical and diagnostic sensitivity and specificity of clinical tests [6,7,8,9,10,11], together greatly expanding the scope and application of liquid biopsies. On the other hand, it promises to shed light on hidden biological processes, which may pave the way for an onrush of new discoveries in human biology.

Among the various epigenetic marks open to interrogation (e.g., DNA methylation and histone modifications), various features relating to the fragmentation profiles of cfDNA molecules are increasingly scrutinized [3,4,12]. These cfDNA features include size signatures [7,11,13,14], preferential cleavage sites [15,16,17], jagged ends [18], unique fragment end-point motifs [19,20], orientation-aware fragmentation patterns [21], nucleosome spacing and density [22,23,24] and topological features such as circular DNA [25,26,27,28]—collectively referred to as fragmentomics analyses. Fragmentomics represents an entirely new modality in the application of cfDNA analysis toward the development of clinically useful liquid biopsy tests and promises to expedite progress in the field.

Here, we focus only on cfDNA size signatures. However, because this is still a young area of inquiry in the field, there are currently many factors that challenge a reliable analysis of cfDNA fragmentation and high-fidelity reconstruction of fragmentomics information in general [1,29], including, but not limited to (i) a lack of knowledge on the biological mechanisms involved in the production of different cfDNA size populations; (ii) overlapping features between pathological and ordinary biological processes; (iii) biological noise induced by the stochastic fluctuations of cfDNA fragmentation features; (iv) unknown preanalytical and physiological variables that impact measurements; (v) significant size bias and variable recovery efficiencies of different cfDNA purification methods; (vi) fragment-length biases of different sequencing chemistries and DNA library preparation methods; (vii) read-length limitations of most DNA sequencing methods; and (viii) resolution limitations and distorted size distributions of widely used capillary electrophoresis methods. Lastly, the characterization of cfDNA fragmentomics signals generated from specific tissues, cell types or mechanisms in vivo is significantly challenged by the co-presence of highly heterogeneous cfDNA populations originating from various other cell and tissue types that dilute the targeted population.

In this work, we address some of the above factors and circumnavigate some drawbacks inherent to in vivo characterization by evaluating the average fragment length and relative proportion of differently sized cfDNA populations isolated from the cell culture supernatant of a human bone osteosarcoma (143B) cell line using different, increasingly sensitive electrophoresis-based DNA sizing methods. The results reveal new insights related to cfDNA fragmentomics and highlight issues that still need to be addressed.

## 2. Materials and Methods

### 2.1. Cell Culturing and Processing of Supernatant

A human bone cancer (osteosarcoma) cell line was acquired from the American Type Culture Collection (143B) (ATCC^®^ CRL-8303™). The cells were grown in Dulbecco’s modified Eagle’s medium (Hyclone DMEM/high glucose) (Thermo Scientific, Waltham, MA, USA; cat# SH30243.01), with 25 mM glucose, 1 mM sodium pyruvate and 4 mM L-glutamine. The culture media were also fortified with 1% penicillin/streptomycin (Lonza, Basel, Switzerland; cat# DE17-602E, lot# 7MB159) and 10% fetal bovine serum (FBS) (PAN Biotech, Aidenbach, Germany; cat# P30-3302). The cells were grown to confluency in 175 cm^2^ cell culture flasks (Thermo Fisher Scientific, Waltham, MA, USA; cat# 159910) under controlled conditions at 37 °C in a humidified atmosphere with 5% CO_2_. The growth medium was then collected from each flask into 50 mL nuclease-free conical tubes (CELLSTAR^®^, Greiner Bio-One, Kremsmünster, Austria; cat# 1882714), centrifuged at 1000× *g* for 10 min at room temperature, and transferred to fresh 50 mL tubes. The supernatant samples were immediately stored at −80 °C until further experiments were performed. The cells were discarded. Control experiments have shown the presence of FBS-derived cfDNA in cell culture supernatant. For this reason, we collected the supernatant only after 28 h of incubation when the cells have reached confluency. Previous studies have shown that cfDNA levels significantly increase over increasing incubation periods [30,31,32], while no FBS-derived DNA was identified in the supernatant of 143B cells collected after 24 h of incubation [33].

### 2.2. Cell-Free DNA Isolation

All cfDNA isolations were performed using the Macherey-Nagel NucleoSpin Gel and PCR Clean-up kit (Macherey-Nagel, Düren, Germany; cat# 740609250) from 2–30 mL of cell culture supernatant. This kit has consistently been used for the characterization of cfDNA in our cell culture studies [30,31,32,33,34], and has shown the best repeatability in a comparative study of six kits [35]. All steps were followed as per the manufacturer’s guidelines stipulated in the PCR clean-up protocol, with minor adaptations made for larger sample volume extractions (as was required to obtain sufficient cfDNA quantities for agarose gel experiments). Briefly, the samples were thawed at 37 °C in a water bath, vortexed, mixed in with binding buffer NTI in a 1:2 ratio and vortexed for 5 s on a medium speed setting. Samples were then added to the spin columns in increments of 600 µL and centrifuged at 11,000× *g* for 1 min at room temperature, until the total sample volume had passed through the column. For the large volume extractions, a vacuum pump connected to a vacuum manifold was used for extraction. Extender tubes were added to the spin columns attached to the manifold and filled (in three increments) with up to a total of 10 mL of cell culture supernatant. The vacuum was then applied until the total sample had passed through the column. After the binding step, the columns were washed twice by the addition of 600 µL wash buffer NT3 and centrifugation at 11,000× *g* for 1 min at room temperature. The columns were dried by centrifugation and cfDNA was eluted into 15 μL elution buffer NE.

### 2.3. Cell-Free DNA Quantification

CfDNA quantification analyses were performed using the Qubit^®^ fluorometer 3.0 (Invitrogen, Life Technologies, Carlsbad, CA, USA) and the Qubit™ dsDNA HS Assay Kit (Invitrogen, Life technologies, Carlsbad, CA, USA; cat# Q32851). All preparations were made according to the manufacturer’s instructions. Briefly, 1–3 µL of cfDNA was added to 197–199 µL of the Qubit working solution, mixed by vortexing and incubated for 2 min at room temperature. Concentrations were calculated from a standard curve.

### 2.4. Cell-Free DNA Size Analysis

#### 2.4.1. Automated Size Analysis of cfDNA

The automated size analysis of cfDNA was performed by means of capillary electrophoresis (CE) using four different assays on two instruments (Table 1). CE utilizes the principles of traditional gel electrophoresis in chip format, and relies on the instrument software to automatically calculate the concentration and size of cfDNA fragments. Dye molecules intercalate into double-stranded DNA molecules and are then detected by laser-induced fluorescence. The relative fluorescence of an internal marker of a known concentration is used to calculate the concentration of differently sized-cfDNA populations within the sample, whereas the size of cfDNA molecules is determined by matching their migration times with the migration times of internal standards of known size.

The extraction, quantification and automated sizing methods used in this study are not suitable for the analysis of single-stranded DNA (ssDNA). Concerning the extraction kit, the protocol using binding buffer NTI is capable of isolating ssDNA, but buffer NTC is recommended for ssDNA, especially for nucleotides shorter than 150 bp. In contrast, the Qubit quantification and Agilent DNA sizing assays utilize an intercalating dye and does therefore not measure ssDNA.

#### 2.4.2. Agarose Gel Electrophoresis

Several different configurations of agarose gel electrophoresis conditions were tested for the optimal separation of nucleosomal cfDNA bands. Variables included gel percentage, gel thickness, well size, buffer type (TBE vs. TAE), the volume of sample loaded, concentration of cfDNA, run time and applied voltage. The following protocol gave the highest resolution separation of cfDNA fragments: 10 cm-long TBE agarose gels (1.5%; 4 mm) were prepared and ran with TBE buffer (1X) for 90 min at 100 V. The staining of the gels was performed using SYBR™ Gold Nucleic Acid Gel Stain (diluted to 1X in TBE; pH level 7–8.5), which is 25–100X more sensitive than traditionally used ethidium bromide, for 40 min in the dark with gentle agitation. A total mass of more than 50 ng cfDNA (not less than 10 ng/µL) in each well gave the best results. Such a high concentration of cfDNA was obtained through two steps. First, we implemented a modified protocol for the extraction of cfDNA from large volumes of cell culture supernatant (see Section 2.2). Second, the concentration of isolated cfDNA was further increased by filtering pooled samples through 30 KDa Amicon Ultra Centrifugal Filter Units by centrifugation at 14,000× *g* for 20 min at room temperature followed by a second centrifugation step at 1000× *g* for 2 min to collect the concentrates. Size analysis of cfDNA using the Fragment Analyzer (FA) dsDNA 930 assays confirmed that no cfDNA size artefacts were generated by the Amicon filter tubes.

### 2.5. Statistics

All statistics were performed using either the GraphPad Prism 9 graphing and statistical software program or Microsoft Excel 2019. Only *p*-values < 0.05 were considered statistically significant.

## 3. Results and Discussion

The purpose of this study was to evaluate the average fragment length and relative proportion of differently sized cfDNA populations isolated from the cell culture supernatant of a human bone osteosarcoma (143B) cell line using different, increasingly sensitive electrophoresis-based DNA sizing methods.

### 3.1. CfDNA Size Profiles as Determined by Different Microfluidic Capillary Electrophoresis Assays

**Figure 1:**
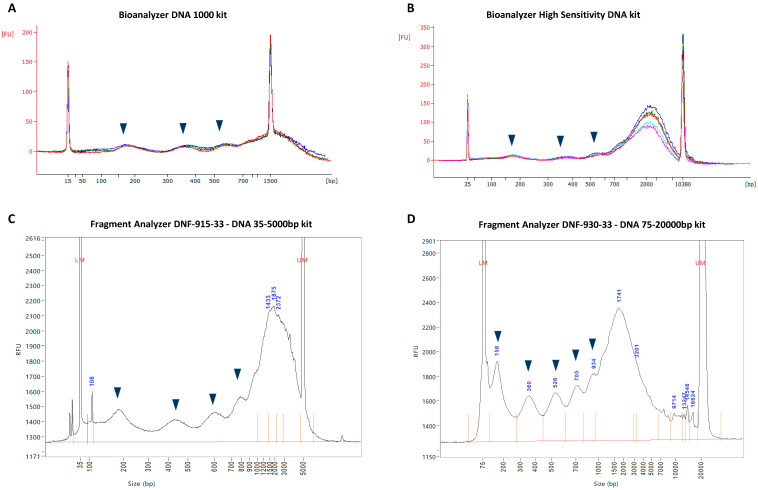
Size profiles of cell-free DNA (cfDNA) isolated from cell culture supernatant. CfDNA was directly isolated from the cell culture supernatant of a human bone osteosarcoma (143B) cell line and analyzed by four different automated microfluidic capillary electrophoresis assays of increasing sensitivity and resolution, including the (**A**) Bioanalyzer DNA 1000 kit; (**B**) Bioanalyzer HS DNA kit; (**C**) FA 915 kit; and (**D**) FA 930 kit. In all of the assays, DNA molecules are detected by laser-induced fluorescence, and the relative fluorescence of the upper marker (*y* axis) is used to calculate the concentration of differently sized-cfDNA populations within the sample. The size of individual cfDNA molecules (*x* axis) is determined by matching their migration times with the migration times of several internal standards of known size. Arrow markers indicate cfDNA populations that correspond to nucleosomal repeats of increasing length. All graphs in this figure are representative of several experimental repeats, i.e., between 2–3 biological repeats and 3–5 technical repeats (see Supplementary Figures S1–S29 for all results).

Size analysis using the Bioanalyzer DNA 1000 kit indicated the presence of four distinct cfDNA populations, including three peaks that correspond to short cfDNA fragment lengths which are characteristic of DNA wound around (i) mono-nucleosomes; (ii) di-nucleosomes; and (iii) tri-nucleosomes; and (iv) one peak of longer cfDNA fragments, the size of which could not be accurately determined due to significant overlapping with the upper marker (Figure 1A). The Bioanalyzer HS DNA assay demonstrated a similar size distribution, but showed the higher resolution sizing of the longer cfDNA size population, which ranged between ~700 and 6000 bp (Figure 1B). The dsDNA 915 assay demonstrated a similar size profile as the Bioanalyzer HS DNA assay, but indicated the presence of an additional cfDNA population that corresponds to a stretch of DNA associated with a chain of four nucleosomes, and demonstrated a narrower estimation of the larger cfDNA size population, ranging between ~900 and 5000 bp (Figure 1C). The dsDNA 930 assay showed a size profile similar to that of the dsDNA 915 assay, but indicated the presence of an additional cfDNA population that corresponds to a stretch of DNA associated with a chain of five nucleosomes, and demonstrated an even narrower estimation of the larger cfDNA size population, ranging between ~1000 and 4000 bp (Figure 1D).

### 3.2. CfDNA Size Profiles as Determined by Agarose Gel Electrophoresis

**Figure 2:**
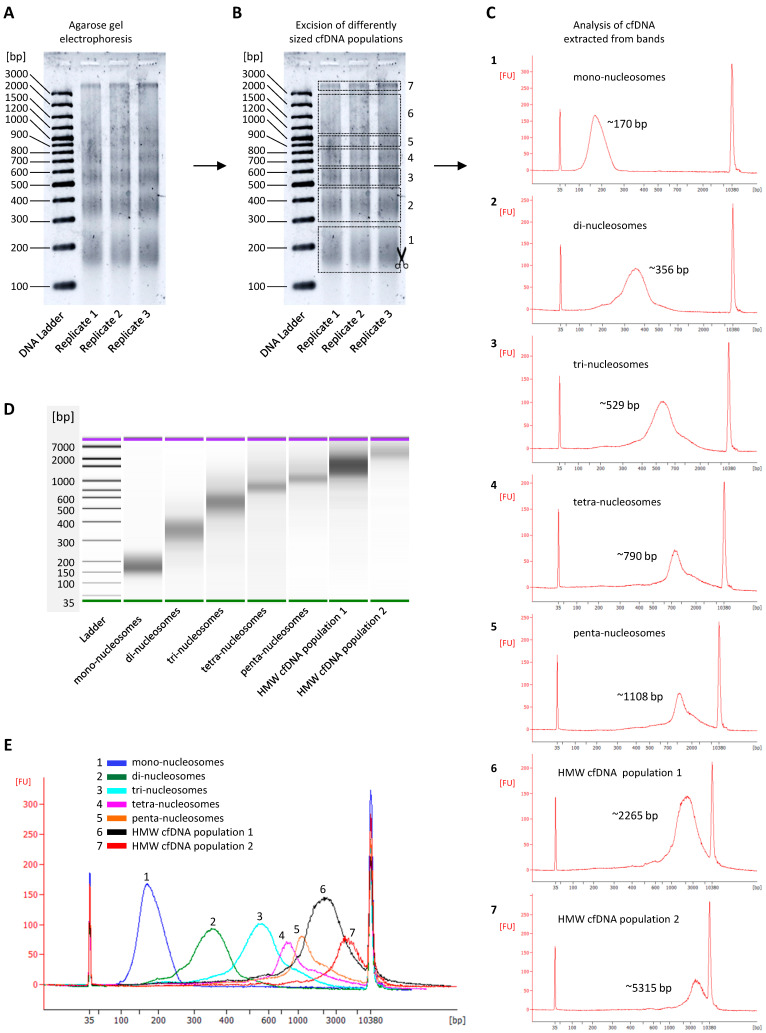
Characterization of individual cell-free DNA (cfDNA) size populations. CfDNA was directly isolated from the cell culture supernatant of a human bone osteosarcoma (143B) cell line. (**A**) After increasing the concentration through a high-volume DNA extraction procedure and subsequent centrifugal filtration of pooled samples with 30 KDa Amicon Ultra Centrifugal Filters, differently sized cfDNA populations were separated by agarose gel electrophoresis. Parameters: three replicates of cfDNA samples were loaded onto a 10 cm-long TBE agarose gel (2%; 4 mm) and run with buffer TBE buffer (1X) for 120 min at 100 V. Each sample well was loaded with 3 µL cfDNA (30 ng in total), 2 µL loading dye and 3 µL H_2_O. Before imaging, gels were stained with SYBR Gold Nucleic Acid Gel Stain for 40 min by gentle agitation. (**B**) Following electrophoresis, seven distinct cfDNA populations were cut out from the gel and isolated. Each of the different cfDNA size populations were then analyzed using the Bioanalyzer High Sensitivity DNA Assay. Figure (**C**) shows the electropherograms of each of the different size populations. Figure (**D**) shows the virtual gel and DNA bands generated by the software, demonstrating a DNA laddering pattern. Figure (**E**) shows the overlapping size profile obtained when all individual populations are superimposed.

The gel electrophoresis profile revealed the presence of at least seven distinct cfDNA size populations (Figure 2A). As indicated in Figure 2B, bands of distinct cfDNA populations were cut out and isolated by a gel-extraction procedure and pooled. The analysis of these individual populations using the Bioanalyzer HS DNA assay indicated the presence of cfDNA populations consisting of nucleosomal multiples, i.e., mono-nucleosomes (Figure 2C1), di-nucleosomes (Figure 2C2), tri-nucleosomes (Figure 2C3), tetra-nucleosomes (Figure 2C4), penta-nucleosomes (Figure 2C5), and two longer cfDNA populations with modal sizes of ~2265 bp (Figure 2C6) and ~5315 bp (Figure 2C7). The visualization of these seven cfDNA populations on a virtual electrophoresis gel demonstrated a clear DNA laddering pattern, which indicates a process of inter-nucleosomal cleavage of DNA (Figure 2D). Furthermore, superimposing these individual peaks onto one electropherogram shows a significant overlap of all cfDNA populations that exceeded the length of three nucleosomes. This indicates that the Bioanalyzer HS DNA assay is not able to reveal the presence of cfDNA subpopulations that are longer than three nucleosomes when they co-occur in one sample (Figure 2E).

**Figure 3:**
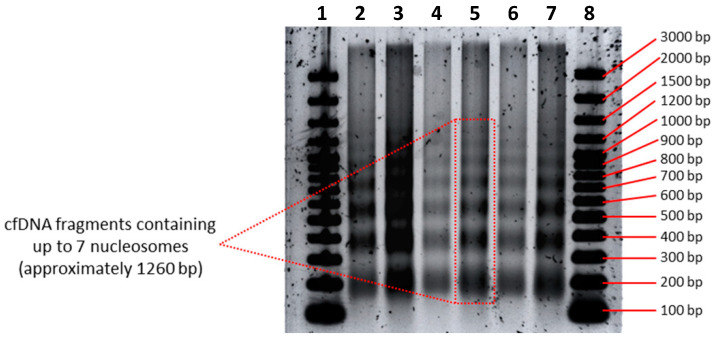
Cell-free DNA (cfDNA) separation and sizing by agarose gel electrophoresis. CfDNA was isolated from large volumes (~30 mL) of human bone osteosarcoma (143B) cell culture supernatant and loaded onto an agarose gel, revealing a clear laddering pattern showing seven distinct cfDNA populations up to seven nucleosomes in length. Wells 1 and 8 contain the GeneRuler 100 bp Plus DNA ladder. Wells 2, 4 and 6 were loaded with 60, 42 and 48 ng cfDNA, respectively (all containing 3 µL cfDNA sample, 2 µL loading dye and 3 µL H_2_O). Wells 3, 5 and 7 were loaded with larger volumes of the same cfDNA samples, amounting to 100, 70 and 80 ng cfDNA, respectively (all containing 5 µL cfDNA sample, 2 µL loading dye and 1 µL H_2_O). Electrophoresis parameters: pooled cfDNA samples concentrated with 30 KDa Amicon Ultra Centrifugal Filter Units were loaded onto a 10 cm-long TBE agarose gel (1.5%; 4 mm) and run with buffer TBE buffer (1X) for 90 min at 100 V. Before imaging, gels were stained with SYBR Gold Nucleic Acid Gel Stain for 40 min by gentle agitation.

To achieve enhanced separation and sizing of longer cfDNA fragments, we evaluated several variations to standard agarose gel electrophoresis procedures in order to establish an optimized protocol (see Section 2.4.2). In comparison with FA assays, this optimized agarose gel electrophoresis approach revealed additional cfDNA size populations consisting of even longer nucleosome chains, including cfDNA fragments consisting of six and seven nucleosomes, respectively (Figure 3). We were not able to further increase the sensitivity of this method to reveal additional bands.

In an internal investigation of cfDNA fragment size, Agilent researchers found that their Femto Pulse system, which is more sensitive than the FA dsDNA 930 assay, also revealed up to seven nucleosomal multiples (see their application note: “cfDNA Separated on the Agilent Femto Pulse System”). Thus, regarding cfDNA analysis, the experimental resolution limit of the most sensitive electrophoresis assays currently seems to be hepta-nucleosomes. In Section 3.5, we discussed whether the DNA laddering pattern continues beyond hepta-nucleosomes, and to which extent.

### 3.3. Resolution Limitations of Electrophoresis-Based cfDNA Sizing

Taken together, the results discussed above firstly demonstrate that different sizing methods generate different cfDNA size profiles. Second, this comparative analysis shows that additional nucleosomal multiples are revealed, and the size range of the longer cfDNA populations narrows correspondingly, as the sensitivity and resolution of the sizing methods increase incrementally. This indicates that each method reaches a resolution limit, where the resolving power is not sufficient for the separation of longer cfDNA fragments. The technical reason for this is that DNA migrates through a gel matrix at a rate that is inversely proportional to its size, which results in short fragments traveling faster than large fragments. This migration, however, does not follow a linear pattern and can rather be described using a logarithmic scale. Therefore, the migration speed of a DNA fragment is determined by the logarithm of its molecular weight, which causes smaller fragments to move even faster and larger fragments to move even slower. This is also why we see the logarithmic distribution of fragment lengths in electrophoretic DNA ladders. It is therefore expected that the electrophoresis methods used in this study are limited in their capacity to adequately separate fragments larger than a specific cut-off with high resolution. Consequently, the relative abundances of each smaller overlapping sub-population within the cluster peaks are impossible to distinguish accurately, resulting in the reporting of a single “large” peak with an overestimated modal size and concentration. This significant overlap of peaks is illustrated in Figure 2E.

A further limitation is that none of the sizing methods used in this study yielded sharp peaks for any of the nucleosomal cfDNA size populations. This indicates that the cfDNA fragments that make up a single peak (representing a nucleosomal multiple) are not all exactly the same size, and that the allocated peak size is only an average. This is expected as the linker DNA stretches that are attached to the nucleosomes can range between 20 and 80 bp in length, which means that a range of fragment sizes are expected for each population (e.g., tri-nucleosomes would be represented by fragment lengths ranging from ~498 bp to 600 bp). The resulting peaks/bands are therefore much broader than those expected for a DNA population of precisely one size (e.g., PCR products or restriction digests), therefore causing significant overlaps, making them even more difficult to distinguish. These overlapping, poorly resolved or entirely unresolved peaks/bands (smears) are then misinterpreted by the sizing software on instruments such as the Bioanalyzer and Fragment Analyzer. Due to the compact distribution and overlapping of the larger fragment size peaks, the software groups several of these smaller peaks into one large peak. The software algorithms are also only capable of assigning accurate sizes and concentrations to the peaks of sufficiently resolved fragment populations, and attempt to do the same for large cluster peaks, thereby labelling the large peaks with sizes that are unrepresentative of the grouped sub-populations [36].

### 3.4. CfDNA Degradation Patterns

The average length of the cfDNA fragments that constitute each of the different cfDNA size populations as determined by dsDNA 930 assay is shown in Table 2. Here, we will discuss the size measurements of one experimental replicate, although similar results were obtained for several experimental repeats (Table 2).

Mono-nucleosomal cfDNA fragments demonstrate a modal size of 157–158 bp. Assuming that approximately 147 bp of this DNA is wound around the nucleosome core particle (NCP) [37], a size of 157–158 bp suggests an average total residual linker DNA of 10–11 bp. This, in turn, indicates the possible involvement of Caspase-activated DNase (CAD) in the inter-nucleosomal cleavage of DNA. When chromatin is digested by CAD, nucleosomal particles of 157 or 158 bp can be formed [38], wherein linker DNA is cleaved 5 or 6 bp away from either side of the 147 bp core particle. However, it is not clear why CAD only sometimes cuts at these sites.

During apoptosis, the digestion of DNA by Caspase-activated DNase (CAD) generally produces ~167 bp mono-nucleosomes, also known as a chromatosome, defined as 147 bp DNA wrapped around the NCP plus 20 bp linker DNA bound to histone H1 [30,31,39]. As CAD has been shown to digest linker DNA in 5–6 bp increments from the NCP, a modal size of 167 bp suggests the cleavage of DNA 10 bp both up and downstream from the NCP [38]. The prevalence of the 167 bp mono-nucleosome structure in the fragment-length distribution of many cfDNA studies indicates that the chromatosome is a stabilizing structure that prevents further enzymatic cleavage of cfDNA molecules in bodily fluids, particularly those in circulation. However, mono-nucleosomes of various sizes have been detected in various biospecimens. The commonly observed 177–178 bp mono-nucleosome [32,39], for example, may be the result of CAD sliding its cleavage site 15 or 16 bp away from the NCP. In the case of our study, the overrepresentation of mono-nucleosomes with an average size of 157–158 bp may suggest a preference for or higher degree of cleavage of linker DNA 5 or 6 bp away from both sides of the 147 bp NCP. The reason for this is not clear and more research is needed to confirm whether CAD is actually involved in the specific conditions of our study. However, there are some interesting lines of indirect evidence that may be considered here:

In a previous cell culture study, we observed time-dependent changes in the modal size of mono-nucleosomes [32]. For example, in the 143B cell line, the modal sizes of cell-free mono-nucleosomes were found to fluctuate over increasing incubation periods, whereas the sizes of mono-nucleosomes in the human dermal microvascular endothelial cell line (HMEC-1) were found to decrease over longer incubation times. Unlike the 143B cells which exhibited a mixed population of differently sized cfDNA populations, HMEC-1 cells were characterized by an overrepresentation of mono-nucleosomes, which simplifies analyses. Interestingly, the ~5 bp stepwise decrease in the length of mono-nucleosomes over longer incubation periods was mirrored by a stepwise increase in H3.1 and H3K27Me3 marks but negatively correlated with a stepwise decrease in H3K14Ac and H4K16Ac marks. This suggests that mono-nucleosomes of longer length (e.g., ~167 bp and ~177 bp) may originate from more loosely packed chromatin regions, while shorter mono-nucleosomes (i.e., ~157–158 bp) may originate from more tightly packed chromatin. The reason why more tightly packed chromatin may produce shorter mono-nucleosomes is not clear, but possible explanations include shorter linker DNA lengths in heterochromatin [40], loss of H1 and linker DNA, or that the stronger binding of nucleosome components and tighter winding of the DNA around the NCP only makes accessible a small segment of the linker DNA to be digested by CAD.

These time-dependent shifts in epigenetic marks and the size of mono-nucleosomes were further mirrored by both changes in the cell cycle, i.e., rapid cell division and increases in the relative proportion of apoptotic and necrotic cell death. This is in line with the observation that both mitosis and apoptotic and necrotic cell death are characterized by the significant condensation of chromatin.

In contrast to mono-nucleosomes, di-, tri-, tetra- and penta-nucleosomes demonstrated modal sizes of 357 bp, 532 bp, 712 bp and 923 bp, respectively (Table 2). Dividing this by the total number of corresponding nucleosomes gives an average length of 178 bp, 177 bp, 178 bp and 184 bp, respectively. As alluded to earlier, it is likely that the size of penta-nucleosomes is slightly overestimated due to the resolution limits of the assay. Thus, an average of 177–178 bp is assumed for each particle in longer nucleosomal chains. Assuming again that an average of 147 bp DNA is associated with the NCP, this suggests that each nucleosome in the chain is associated with linker DNA of approximately 30–31 bp. Unlike mono-nucleosomes with a modal size of ~157–158 bp which can only be produced by the digestion of linker DNA 5 or 6 bp up or- downstream from the NCP, the longer oligo-nucleosomes appear to be generated through a less specific digestion scheme.

**Figure 4:**
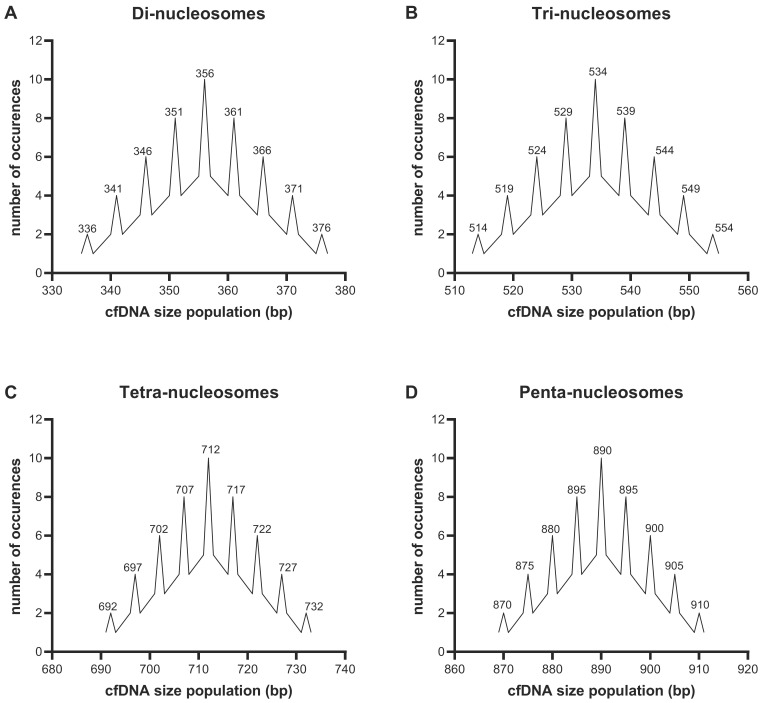
Theoretical cell-free DNA (cfDNA) fragmentation profiles. Here, we calculated the relative proportion of different (**A**) di-nucleosomal, (**B**) tri-nucleosomal, (**C**) tetra-nucleosomal and (**D**) penta-nucleosomal size populations that can theoretically form when oligo-nucleosomes with an average linker DNA length of 31 bp have an equal probability of being digested 5, 6, 10, 11, 15 and 16 bp up- or downstream from a 147 bp nucleosome core particle (NCP).

By calculating the theoretical nucleosome size profile that would form under different sets of conditions (i.e., variation in linker DNA lengths, linker DNA cut-sites and NCP sizes), the specific modal sizes observed in this study best match a digestion scheme in which the ~31 bp linker DNA between oligo-nucleosomes have an equal probability of being digested 5, 6, 10, 11, 15, or 16 bp up or downstream from the ~147 bp NCP (Figure 4). However, bearing in mind that the modal size measurements calculated by the dsDNA 930 assay are not sufficiently sensitive to calculate the size to the exact bp length (as discussed in Section 3.3), especially for longer cfDNA fragment lengths, the cfDNA size profiles only roughly match the probability calculations for the theoretical sizes. Further experiments, thorough mathematical modeling and computer simulations are needed before a conclusion can be concretely drawn.

### 3.5. The Relative Contribution of Differently Sized cfDNA Populations

**Figure 5:**
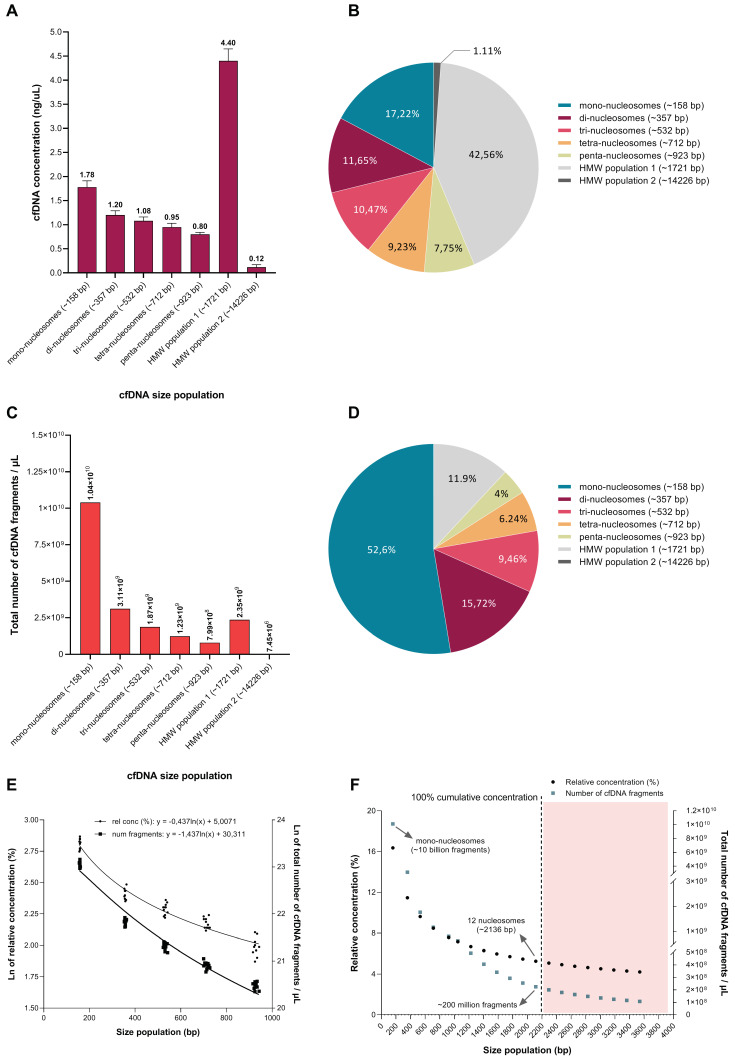
Characterization of differently sized cell-free DNA (cfDNA) populations as determined by the dsDNA 930 assay. Summary of the (**A**) average concentration, (**B**) relative contribution (%) in terms of concentration, (**C**) copy number (total number of cfDNA fragments) frequency distribution and (**D**) relative contribution (%) in terms of the copy number of the differently sized cfDNA populations present in the cell culture supernatant as determined by the dsDNA 930 assay. The total number of individual fragments that constitute each of the differently sized cfDNA populations (copy number) was determined as follows: (i) calculate the total number of base pairs that constitute each size population by dividing the average concentration of the population by the theoretical weight of one base pair (650 Da = 1.67 × 10 − 24 g); and (ii) divide the total number of base pairs by the corresponding modal length of the population. (**E**) Logarithmic equations obtained by the relative concentration (%) (left *y* axis) and total number of cfDNA fragments (right *y* axis), respectively, of the different size populations in several tested samples (*n* = 11) were used to (**F**) forecast the average length (left *y* axis) and total number of fragments (right *y* axis) that constitute the largest nucleosome population that may be present in the samples measured in this study.

In terms of the absolute concentration of differently sized cfDNA populations as determined by the dsDNA 930 assay, the larger ~1721 bp cfDNA population is significantly overrepresented, constituting 42.56% of the total population (Figure 5A). High molecular weight (HMW) cfDNA fragments (>10 Kbp) contribute only 1.11% to the total population. Mono-, di-, tri-, tetra- and hepta-nucleosomes contributed 17.22%, 11.65%, 10.47%, 9.23% and 7.75% toward the total population, respectively, showing a decline in concentration as the length of the DNA chain increases (Figure 5B). In terms of copy number, which refers to the total number of individual cfDNA fragments that comprise each cfDNA size population (Figure 5C), mono-nucleosomes are significantly overrepresented, constituting approximately 52.6% of the total population (Figure 5D). The remainder of the cfDNA population consists of 15.72% di-nucleosomes, 9.46% tri-nucleosomes, 6.24% tetra-nucleosomes, 4.04% penta-nucleosomes, 11.91% longer fragments (~1721 bp) and only 0.04% of fragments longer than 10 Kbp (Figure 5D).

As alluded to earlier, however, the values measured for the longer ~1721 bp cfDNA population should be interpreted with caution as the dsDNA 930 assay is not capable of resolving cfDNA subpopulations that range in size between 1 and 5 Kbp, especially when these subpopulations differ in size by nucleosomal increments and are present in one sample. This suggests that the concentration of cfDNA fragments with a modal size of ~1721 bp is overestimated while the modal size is an artefact. Instead, the ~1721 bp peak represents several oligo-nucleosomal peaks in a DNA laddering pattern which continues beyond penta-nucleosomes, and wherein each successive size population, which consists of one additional nucleosome, has a lower concentration and constitutes less fragments than the previous population. A hypothetical illustration of such a corrected frequency distribution is shown in Figure 6 of ref. [32]. This hypothesis is supported by two observations made in this study: First, increasingly sensitive sizing methods revealed the presence of more nucleosomal multiples (extended DNA laddering pattern) (Figure 1, Figure 2 and Figure 3). Second, the quantitative analysis of differently sized cfDNA populations via the excision of individual agarose gel electrophoresis bands followed by sizing and quantitative analysis, which allows a much more direct assessment of true cfDNA quantity, demonstrated results that more closely fit the expectations, i.e., much lower levels of long cfDNA fragments (Supplementary Figure S30). Using the latter method, the relative contribution (in terms of copy number) of mono-nucleosomes, di-nucleosomes, tri-nucleosomes, tetra-nucleosomes and penta-nucleosomes, HMW population 1 (~2265 bp) and HMW population 2 (~5315 bp) were estimated as 61,5%, 22,3%, 9,9%, 2,4%, 1,4%, 2,1% and 0,3%, respectively (Supplementary Figure S30D). These values are also clearly reflected by the fluorescence intensity of the cfDNA bands (bright for mono-nucleosomes and declining for every successive band) (Figure 2A and Figure 3).

Therefore, the frequency distribution curve of the differently sized cfDNA populations shows an exponential decay, which is characteristic of a power-law distribution (Figure 5C and Supplementary Figure S30C). In terms of the process of inter-nucleosomal DNA cleavage, this may indicate the involvement of a stochastic process: as we have shown in this study, cfDNA molecules represent DNA molecules that are digested into multiple fragments with lengths that generally correspond to integer multiples of a modal length of 160–200 bp, the size of DNA wrapped around a single nucleosome (plus a stretch of linker DNA), thereby generating a DNA “ladder” pattern. In a stochastic cleavage process, wherein (i) all inter-nucleosomal linker DNA sites are roughly equally vulnerable to cleavage, regardless of the length of DNA fragments (i.e., number of nucleosomes in the chain); (ii) cleavage sites are “chosen” at random over time; and (iii) the size and number of cfDNA fragments are sufficiently large, the distribution of fragment lengths will tend toward the form of a power law relation over time.

This means that (1) each cfDNA size population is fed by the degradation of all size populations larger than itself; (2) a relative change in longer nucleosome chains will result in a proportional change in shorter nucleosome chains; and (3) shorter fragments will appear more often. In this study, we did not experimentally investigate which cellular processes may be involved in the generation of this laddering pattern. In a previous study, however, we have shown that both apoptosis and necrosis govern the release of cfDNA from 143B cells under normal physiological conditions, but that necrosis contributes slightly more [32]. We have further shown that the ratio in the concentration of long-to-short cfDNA fragments decrease over increasing incubation periods, suggesting that long fragments are degraded over time [32].

To investigate the extent to which the DNA laddering pattern continues, we extrapolated the exponential decay in the relative contribution of increasingly longer nucleosome populations. We obtained a logarithmic equation by plotting the natural logarithm of the relative concentration (%) of mono-, di-, tri-, tetra- and penta-nucleosomes toward the total population, as determined by the dsDNA 930 assay for two biological replicates and 11 technical replicates (Figure 5E). This log equation was used to forecast the relative percentage contribution of increasingly longer nucleosome populations (Figure 5F). The nucleosome population with the theoretical maximum length was estimated as 12 nucleosomes (~2136 bp), which we defined here as the last nucleosome size population in the extrapolation curve that does not exceed an accumulative concentration percentage of 100%. In other words, the extrapolation curve was generated from the known concentration of different size populations (the first five nucleosomal multiples) as measured by the dsDNA 930 assay. If the extrapolation curve is then used to estimate the concentration of the “unknown” size populations (i.e., hexa-nucleosomes and onward), the cumulative concentration off all fragments up to ~2136 bp would not exceed the total cfDNA concentration measured by the dsDNA 930 assay. According to this model, the presence of cfDNA fragments larger than approximately ~2136 bp would then exceed the total concentration measured by the dsDNA 930 assay. Thus, we hypothesize that the largest cfDNA population of meaningful concentration in our specific samples is ~2136 bp. Using the same approach, we extrapolated the total number of cfDNA fragments that constitute each size population and estimated that the 12-nucleosome size population consists of ~200 million fragments/µL, whereas the mono-nucleosome population consists of ~10 billion fragments/µL (Figure 5F). Arrays containing 12 nucleosomes correspond with a secondary chromatin structure and may represent the “30 nm fiber”, which is a structure characterized by the addition of H1 and the coiling of the “beads-on-a-string” structure into a helical structure with a diameter of 30 nm, and is thought to be the form of heterochromatin [41]. However, the exact structure of this 30 nm fiber is still not resolved, with many possible models still under consideration. Moreover, its biological relevance is questioned by many researchers as nucleosomes seem to be more flexible than initially thought (reviewed in [42]). It is interesting to note, however, that cfDNA populations with this median size have been observed in several cell culture experiments, including in the absence of shorter fragments [30,31,32,43,44], and has been shown to be mainly comprised of repetitive DNA [33].

In this study, the results seem to indicate that a cfDNA population consisting of an array of 12 nucleosomes may serve as the precursor for the generation of shorter cfDNA populations, and that all cfDNA fragments in the sample may share a common origin. More insight into the results presented in this work may be gained by isolating the respective size populations, followed by sequence analysis of each individual cfDNA population.

### 3.6. Limitations of this Study and Future Prospects

CfDNA fragmentation profiles were investigated under specific conditions, i.e., the use of only one cell line, one extraction kit and one DNA sizing principle. While some insights from this study may be transferrable to other cell lines and in vivo conditions, more research is needed to encapsulate the wide-ranging factors that can affect cfDNA size profiling. For example, (i) recent experiments have shown various recovery efficiencies and size bias for the capture of cfDNA from cell culture supernatant [35]; and (ii) cfDNA profiles may differ significantly between different cell lines when measured under baseline conditions, depending on their unique biology (e.g., growth rate, cell death rate and preferred cell death mechanisms) [31,32]. In line with this, cfDNA profiles depend on the time-point (incubation period) at which the supernatant is collected, which reflects differences in confluence, cell viability, nutrient availability, etc. [30,31,32]. To accommodate all of these variables, it would be interesting to use various sizing methods to profile cfDNA isolated by different methods from the supernatant of different cell lines collected at increasing incubation periods, under both normal physiological conditions and stimulation (e.g., the induction of cell death and genomic instability). Furthermore, more insights into cfDNA size profiles may be gained through the use of non-electrophoretic sizing methods. One option is the use of RT-qPCR; however, as PCR amplicons have defined lengths, it is not typically used for accurate sizing but rather to calculate the ratio of the concentration of cfDNA fragments in defined size ranges. Moreover, while the maximum amplicon length tested by companies is approximately 5 Kbp, no cfDNA studies have to date used qPCR to size cfDNA fragments that exceed even 1 Kbp. Even with an upper-limit of 5 Kbp, RT-qPCR is not a suitable method for the accurate profiling of a total cfDNA population, which can range between 0 and 30 Kbp. RT-qPCR is also limited by GC-content that affects efficiency. Another option is the use of DNA sequencing, in particular, Oxford Nanopore sequencing which allows the sequencing of much longer DNA fragments than typical sequencing methods [45,46,47]. Lastly, various other preanalytical variables that may affect cfDNA size profiling need to be considered., e.g., the presence of FBS-derived DNA, shearing during sample handling, the effect of storage temperatures and sample thawing [29,48].

## 4. Conclusions

In this study, we analyzed the fragmentation patterns of cfDNA isolated from the cell culture supernatant of 143B cells using increasingly sensitive electrophoresis methods, including (from least to most sensitive): (i) the Bioanalyzer DNA 1000 kit; (ii) the Bioanalyzer HS DNA kit; (iii) the FA dsDNA 915 kit; (iv) the FA dsDNA 930 kit; and lastly (v) an optimized agarose gel electrophoresis method. This direct comparison generated several interesting observations.

By analyzing samples from the same original source, the least sensitive sizing method revealed only three discernible cfDNA size populations, while the most sensitive method was able to distinguish seven different size populations. Interestingly, the incremental uncovering of additional nucleosomal multiples by increasingly sensitive methods was mirrored by a decrease in the estimation of the size range of HMW cfDNA. Furthermore, the dsDNA 930 assay revealed an exponential decay in the relative contribution of increasingly longer nucleosomal multiples in the cfDNA population, suggesting the involvement of a stochastic inter-nucleosomal DNA cleavage process. In this process, shorter populations accumulate rapidly as they are fed by the degradation of all larger populations, resulting in a significant overrepresentation of mono-nucleosomes. However, this is currently a hypothesis, as more experimental research on in vitro degradation kinetics supported by the mathematical modeling of inter-nucleosomal DNA cleavage under various simulated conditions is needed to confirm whether all linker DNA sites are equally prone to cleavage and are truly “chosen” at random in the specific conditions of this study. In line with this, it is not suggested here that cfDNA degradation always occurs in a stochastic manner, and that there are no preferential cleavage sites or protected regions, which may depend on various factors such as tissue type, cell type and genomic origin of cfDNA fragments (e.g., heterochromatin vs. euchromatin).

Taken together, these results have several implications. First, it demonstrates the significant variation in the resolving capacities of similar electrophoresis-based methods for the sizing of cfDNA. Not only do electrophoresis-based methods have difficulty in separating nucleosomal multiples, but they also do not possess the resolving power to give insight into minor changes in the structure of nucleosomal sub-populations, especially when these populations are co-present in one sample. This highlights the importance of selecting the appropriate cfDNA sizing methods, whether applied as a method in basic research or as a tool for monitoring the quality control in experiments and clinical assays. Second, it indicates that the cfDNA laddering pattern extends well beyond the tri-nucleosomal cfDNA size profile that is typically generated by the most commonly used electrophoresis-based cfDNA sizing method (the Bioanalyzer HS DNA kit). Third, the modal sizes of HMW cfDNA populations are exaggerated due to the limited resolving power of electrophoresis, and instead consist of several poly-nucleosomal subpopulations that continue the series of DNA laddering.

While it was not directly investigated in this study, it seems likely that a significant portion of short and longer cfDNA fragments might share overlapping origins. This may explain why similar size profiles have historically been reported for cfDNA populations originating from different processes, such as apoptosis, necrosis, accidental cell lysis and purported active release. Moreover, while not observed in this study due to the limited resolving capacity of the methods, other researchers have observed sub-nucleosomal fractions of cfDNA. This first seems to suggest that the view that mono-nucleosomal DNA is only produced by apoptosis and is the most important cfDNA fraction is a biased one, and secondly that the notion that all longer cfDNA fragments are genomic contaminations and do not contain valuable biological information needs to be reassessed.

## Figures and Tables

**Table 1:** Automated DNA sizing methods used in this study.

Instrument	Kit	Separation Range (bp)	Input Concentration Range
Agilent 2100 Bioanalyzer	DNA 1000 kit	25–1000 bp	0.5–50 ng/µL
	High Sensitivity (HS) DNA Kit	50–7000 bp	5–500 pg/µL
Agilent Fragment Analyzer	dsDNA 915 Reagent kit (35–5000 bp)	35–5000 bp	0.5–50 ng/µL
	dsDNA 930 Reagent kit (75–20,000 bp)	75–20,000 bp	0.5–50 ng/µL

**Table 2:** The modal length (bp) of differently sized cell-free DNA (cfDNA) populations as determined by the FA dsDNA 930 assay.

Size Population	Biol Rep 1 (Culture Flask 1)				Biol Rep 2 (Culture Flask 2)				All Reps	
	Extr Rep 1		Extr Rep 2		Extr Rep 1		Extr Rep 2			
	Avg	Stdev	Avg	Stdev	Avg	Stdev	Avg	Stdev	Avg	Stdev
Mono-nucleosomes	157.60	0.89	158.00	1.41	158.00	1.41	158.00	1.41	157.90	0.20
Di-nucleosomes	356.80	3.03	350.80	5.81	355.00	2.00	350.40	4.51	353.25	3.15
Tri-nucleosomes	532.40	5.13	529.40	4.22	532.80	6.83	528.80	5.76	530.85	2.04
Tetra-nucleosomes	712.40	7.89	701.00	5.66	714.20	10.62	704.00	8.89	707.90	6.40
Penta-nucleosomes	923.40	5.22	913.33	15.95	930.40	8.05	902.50	40.31	917.41	12.16
HMW population 1	1721.00	14.14	1714.00	89.56	1736.80	42.99	1684.80	97.12	1714.15	21.77
HMW population 2	14,226.24	2283.82	13,181.24	3196.39	14,034.72	2449.88	12,657.88	3738.73	13,525.02	735.22

For each cell culture flask, DNA was extracted in duplicate. For each DNA extraction, at least 5 technical replicates were analyzed by the FA dsDNA 930 assay. Thus, the average size in each column represents the average size determined for at least 5 technical replicates. Abbreviations: Biol rep: Biological replicate; Extr rep: Extraction replicate; Avg: Average; Stdev: Standard deviation.

## Data Availability

Not applicable.

## Supplementary Materials

The following supporting information can be downloaded at: https://www.mdpi.com/article/10.3390/diagnostics12081896/s1, Supplementary Figures: Experimental replicates and supporting experiments.

## References

[1] van der Pol Y., Mouliere F. (2019). Toward the Early Detection of Cancer by Decoding the Epigenetic and Environmental Fingerprints of Cell-Free DNA. Cancer Cell.

[2] Bronkhorst A.J., Ungerer V., Diehl F., Anker P., Dor Y., Fleischhacker M., Gahan P.B., Hui L., Holdenrieder S., Thierry A.R. (2021). Towards systematic nomenclature for cell-free DNA. Human Genet..

[3] Lo Y.D., Han D.S., Jiang P., Chiu R.W. (2021). Epigenetics, fragmentomics, and topology of cell-free DNA in liquid biopsies. Science.

[4] Ding S.C., Lo Y.D. (2022). Cell-Free DNA Fragmentomics in Liquid Biopsy. Diagnostics.

[5] Bronkhorst A.J., Ungerer V., Holdenrieder S. (2019). The emerging role of cell-free DNA as a molecular marker for cancer management. Biomol. Detect. Quantif..

[6] Vessies D.C., Schuurbiers M.M., van der Noort V., Schouten I., Linders T.C., Lanfermeijer M., Ramkisoensing K.L., Hartemink K.J., Monkhorst K., van den Heuvel M.M. (2022). Combining variant detection and fragment length analysis improves detection of minimal residual disease in post-surgery circulating tumour DNA of stage II-IIIA NSCLC patients. Mol. Oncol..

[7] Mouliere F., Chandrananda D., Piskorz A.M., Moore E.K., Morris J., Ahlborn L.B., Mair R., Goranova T., Marass F., Heider K. (2018). Enhanced detection of circulating tumor DNA by fragment size analysis. Sci. Transl. Med..

[8] Underhill H.R. (2021). Leveraging the fragment length of circulating tumour DNA to improve molecular profiling of solid tumour malignancies with next-generation sequencing: A pathway to advanced non-invasive diagnostics in precision oncology?. Mol. Diagn. Ther..

[9] Zviran A., Schulman R.C., Shah M., Hill S.T., Deochand S., Khamnei C.C., Maloney D., Patel K., Liao W., Widman A.J. (2020). Genome-wide cell-free DNA mutational integration enables ultra-sensitive cancer monitoring. Nat. Med..

[10] Chabon J.J., Hamilton E.G., Kurtz D.M., Esfahani M.S., Moding E.J., Stehr H., Schroers-Martin J., Nabet B.Y., Chen B., Chaudhuri A.A. (2020). Integrating genomic features for non-invasive early lung cancer detection. Nature.

[11] Markus H., Chandrananda D., Moore E., Mouliere F., Morris J., Brenton J.D., Smith C.G., Rosenfeld N. (2022). Refined characterization of circulating tumor DNA through biological feature integration. Sci. Rep..

[12] Chiu R.W., Heitzer E., Lo Y.D., Mouliere F., Tsui D.W. (2020). Cell-free DNA fragmentomics: The new “Omics” on the block. Clin. Chem..

[13] Sanchez C., Snyder M.W., Tanos R., Shendure J., Thierry A.R. (2018). New insights into structural features and optimal detection of circulating tumor DNA determined by single-strand DNA analysis. NPJ Genom. Med..

[14] Underhill H.R., Kitzman J.O., Hellwig S., Welker N.C., Daza R., Baker D.N., Gligorich K.M., Rostomily R.C., Bronner M.P., Shendure J. (2016). Fragment Length of Circulating Tumor DNA. PLoS Genet..

[15] Chandrananda D., Thorne N.P., Bahlo M. (2015). High-resolution characterization of sequence signatures due to non-random cleavage of cell-free DNA. BMC Med. Genom..

[16] Chan K.A., Jiang P., Sun K., Cheng Y.K., Tong Y.K., Cheng S.H., Wong A.I., Hudecova I., Leung T.Y., Chiu R.W. (2016). Second generation noninvasive fetal genome analysis reveals de novo mutations, single-base parental inheritance, and preferred DNA ends. Proc. Natl. Acad. Sci. USA.

[17] Sun K., Jiang P., Wong A.I.C., Cheng Y.K.Y., Cheng S.H., Zhang H., Chan K.C.A., Leung T.Y., Chiu R.W.K., Lo Y.M.D. (2018). Size-tagged preferred ends in maternal plasma DNA shed light on the production mechanism and show utility in noninvasive prenatal testing. Proc. Natl. Acad. Sci. USA.

[18] Jiang P., Xie T., Ding S.C., Zhou Z., Cheng S.H., Chan R.W., Lee W.-S., Peng W., Wong J., Wong V.W. (2020). Detection and characterization of jagged ends of double-stranded DNA in plasma. Genome Res..

[19] Jiang P., Sun K., Tong Y.K., Cheng S.H., Cheng T.H., Heung M.M., Wong J., Wong V.W., Chan H.L., Chan K.A. (2018). Preferred end coordinates and somatic variants as signatures of circulating tumor DNA associated with hepatocellular carcinoma. Proc. Natl. Acad. Sci. USA.

[20] Serpas L., Chan R.W.Y., Jiang P., Ni M., Sun K., Rashidfarrokhi A., Soni C., Sisirak V., Lee W.-S., Cheng S.H. (2018). Dnase1l3 deletion causes aberrations in length and end-motif frequencies in plasma DNA. Proc. Natl. Acad. Sci. USA.

[21] Sun K., Jiang P., Cheng S.H., Cheng T.H.T., Wong J., Wong V.W.S., Ng S.S.M., Ma B.B.Y., Leung T.Y., Chan S.L. (2019). Orientation-aware plasma cell-free DNA fragmentation analysis in open chromatin regions informs tissue of origin. Genome Res..

[22] Snyder M.W., Kircher M., Hill A.J., Daza R.M., Shendure J. (2016). Cell-free DNA Comprises an In Vivo Nucleosome Footprint that Informs Its Tissues-Of-Origin. Cell.

[23] Ulz P., Perakis S., Zhou Q., Moser T., Belic J., Lazzeri I., Wolfler A., Zebisch A., Gerger A., Pristauz G. (2019). Inference of transcription factor binding from cell-free DNA enables tumor subtype prediction and early detection. Nat. Commun..

[24] Ulz P., Thallinger G.G., Auer M., Graf R., Kashofer K., Jahn S.W., Abete L., Pristauz G., Petru E., Geigl J.B. (2016). Inferring expressed genes by whole-genome sequencing of plasma DNA. Nat. Genet..

[25] Sin S.T., Jiang P., Deng J., Ji L., Cheng S.H., Dutta A., Leung T.Y., Chan K.A., Chiu R.W., Lo Y.D. (2020). Identification and characterization of extrachromosomal circular DNA in maternal plasma. Proc. Natl. Acad. Sci. USA.

[26] Zhu J., Zhang F., Du M., Zhang P., Fu S., Wang L. (2017). Molecular characterization of cell-free eccDNAs in human plasma. Sci. Rep..

[27] Kumar P., Dillon L.W., Shibata Y., Jazaeri A.A., Jones D.R., Dutta A. (2017). Normal and Cancerous Tissues Release Extrachromosomal Circular DNA (eccDNA) into the Circulation. Mol. Cancer Res..

[28] Grabuschnig S., Bronkhorst A.J., Holdenrieder S., Rosales Rodriguez I., Schliep K.P., Schwendenwein D., Ungerer V., Sensen C.W. (2020). Putative Origins of Cell-Free DNA in Humans: A Review of Active and Passive Nucleic Acid Release Mechanisms. Int. J. Mol. Sci..

[29] van der Pol Y., Moldovan N., Verkuijlen S., Ramaker J., Boers D., Onstenk W., de Rooij J., Bahce I., Pegtel D.M., Mouliere F. (2022). The Effect of Preanalytical and Physiological Variables on Cell-Free DNA Fragmentation. Clin. Chem..

[30] Bronkhorst A.J., Wentzel J.F., Aucamp J., van Dyk E., du Plessis L., Pretorius P.J. (2016). Characterization of the cell-free DNA released by cultured cancer cells. BBA-Mol Cell Res..

[31] Aucamp J., Bronkhorst A.J., Peters D.L., Van Dyk H.C., Van der Westhuizen F.H., Pretorius P.J. (2017). Kinetic analysis, size profiling, and bioenergetic association of DNA released by selected cell lines in vitro. Cell. Mol. Life Sci..

[32] Ungerer V., Bronkhorst A.J., Van den Ackerveken P., Herzog M., Holdenrieder S. (2021). Serial profiling of cell-free DNA and nucleosome histone modifications in cell cultures. Sci. Rep..

[33] Bronkhorst A.J., Wentzel J.F., Ungerer V., Peters D.L., Aucamp J., de Villiers E.P., Holdenrieder S., Pretorius P.J. (2018). Sequence analysis of cell-free DNA derived from cultured human bone osteosarcoma (143B) cells. Tumour Biol..

[34] Bronkhorst A.J., Ungerer V., Holdenrieder S. (2019). Comparison of methods for the quantification of cell-free DNA isolated from cell culture supernatant. Tumor Biol..

[35] Bronkhorst A.J., Ungerer V., Holdenrieder S. (2020). Comparison of methods for the isolation of cell-free DNA from cell culture supernatant. Tumor Biol..

[36] Ziraldo R., Shoura M.J., Fire A.Z., Levene S.D. (2019). Deconvolution of nucleic-acid length distributions: A gel electrophoresis analysis tool and applications. Nucleic Acids Res..

[37] Korolev N., Lyubartsev A.P., Nordenskiöld L. (2018). A systematic analysis of nucleosome core particle and nucleosome-nucleosome stacking structure. Sci. Rep..

[38] Allan J., Fraser R.M., Owen-Hughes T., Keszenman-Pereyra D. (2012). Micrococcal nuclease does not substantially bias nucleosome mapping. J. Mol. Biol..

[39] Shi J., Zhang R., Li J., Zhang R. (2020). Size profile of cell-free DNA: A beacon guiding the practice and innovation of clinical testing. Theranostics.

[40] Makarov V.L., Lejnine S., Bedoyan J., Langmore J.P. (1993). Nucleosomal organization of telomere-specific chromatin in rat. Cell.

[41] Tremethick D.J. (2007). Higher-order structures of chromatin: The elusive 30 nm fiber. Cell.

[42] Bonev B., Cavalli G. (2016). Organization and function of the 3D genome. Nat. Rev. Genet..

[43] Aucamp J., Van Dyk H.C., Bronkhorst A.J., Pretorius P.J. (2017). Valproic acid alters the content and function of the cell-free DNA released by hepatocellular carcinoma (HepG2) cells in vitro. Biochimie.

[44] Panagopoulou M., Karaglani M., Balgkouranidou I., Pantazi C., Kolios G., Kakolyris S., Chatzaki E. (2019). Circulating cell-free DNA release in vitro: Kinetics, size profiling, and cancer-related gene methylation. J. Cell. Physiol..

[45] Yu S.C., Jiang P., Peng W., Cheng S.H., Cheung Y.T., Tse O.O., Shang H., Poon L.C., Leung T.Y., Chan K.A. (2021). Single-molecule sequencing reveals a large population of long cell-free DNA molecules in maternal plasma. Proc. Natl. Acad. Sci. USA.

[46] Cheng S.H., Jiang P., Sun K., Cheng Y.K., Chan K.A., Leung T.Y., Chiu R.W., Lo Y.D. (2015). Noninvasive prenatal testing by nanopore sequencing of maternal plasma DNA: Feasibility assessment. Clin. Chem..

[47] Lang D., Zhang S., Ren P., Liang F., Sun Z., Meng G., Tan Y., Li X., Lai Q., Han L. (2020). Comparison of the two up-to-date sequencing technologies for genome assembly: HiFi reads of Pacific Biosciences Sequel II system and ultralong reads of Oxford Nanopore. Gigascience.

[48] Bronkhorst A.J., Aucamp J., Pretorius P.J. (2015). Cell-free DNA: Preanalytical variables. Clin. Chim. Acta.

