# Supplementary material for: Cell-Free DNA Fragmentation Patterns in a Cancer Cell Line
Source: Diagnostics (Basel). 2022 Aug 4;12(8):1896. doi: 10.3390/diagnostics12081896 (PMC9406536; doi:10.3390/diagnostics12081896)
Supplement: Supplementary file 1 [file diagnostics-12-01896-s001.zip › diagnostics-1776561-supplementary.pdf]

Supplementary Figures: Experimental replicates and supporting experiments

Supplementary figures representing the results of the **Fragment Analyzer DNF-930-33 (75-20000 bp)** assays. (S1) Ladder; (S2-S7) Biological replicate 1; and (S8-S12) Biological replicate 2.

S1

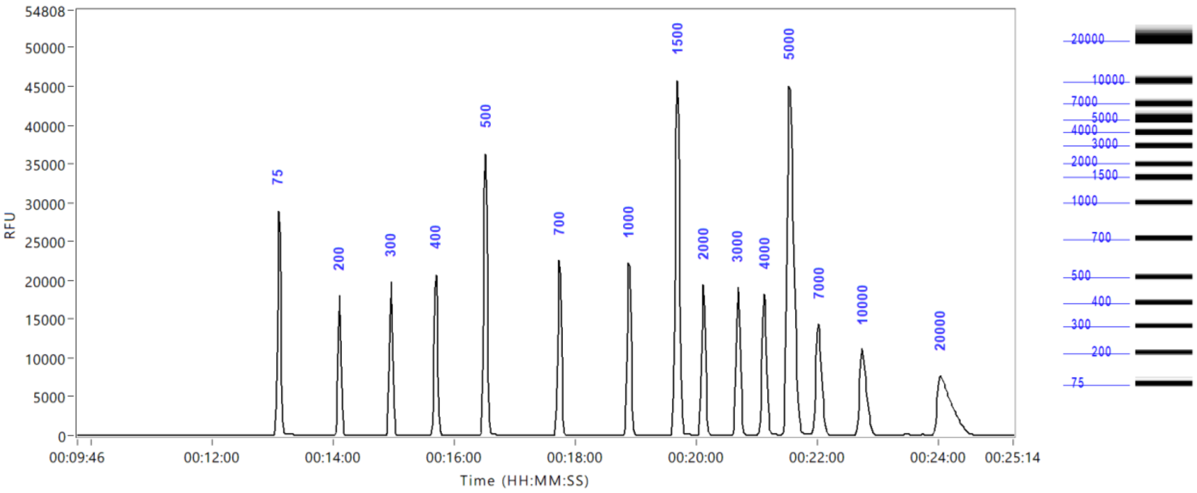

S2

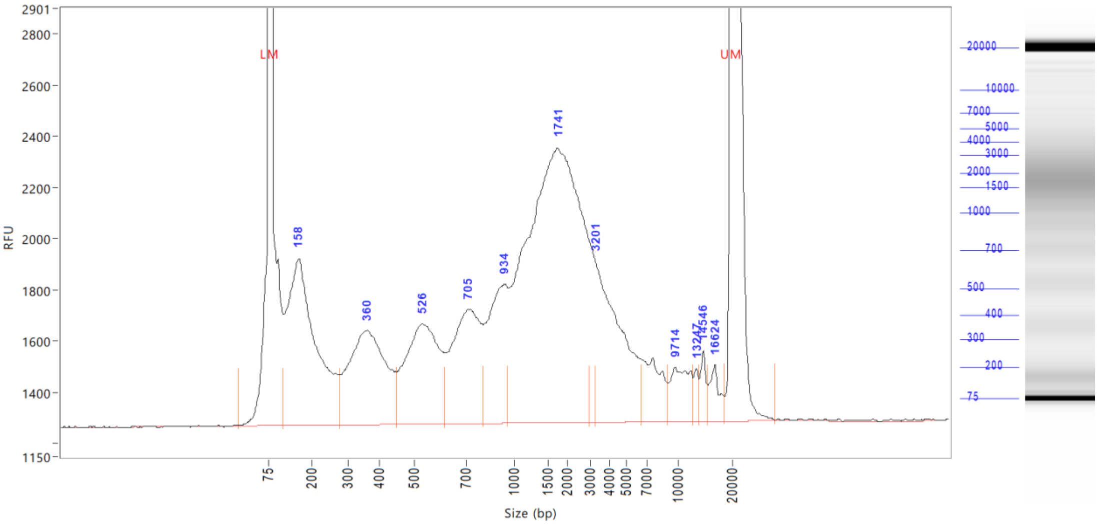

S3

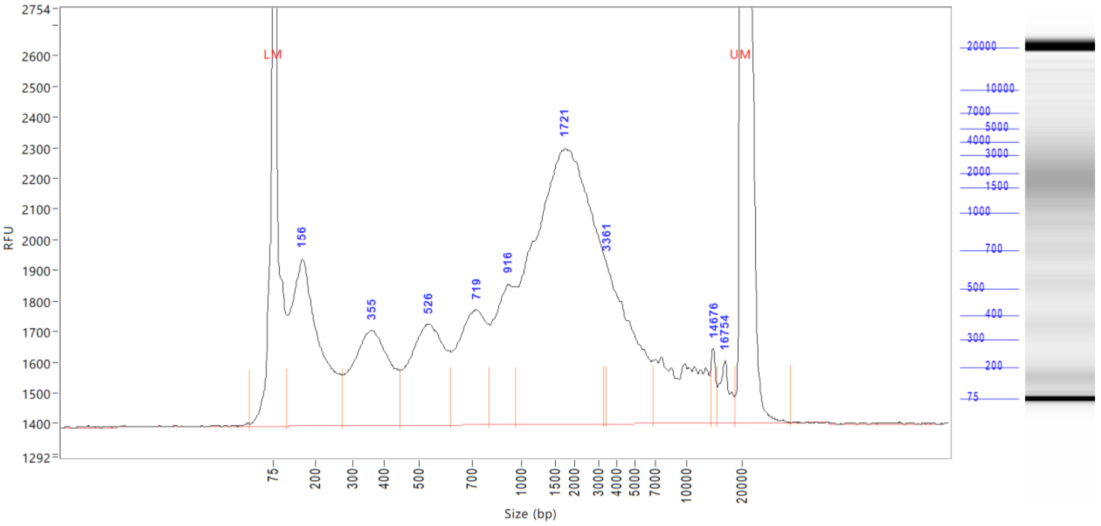

S4

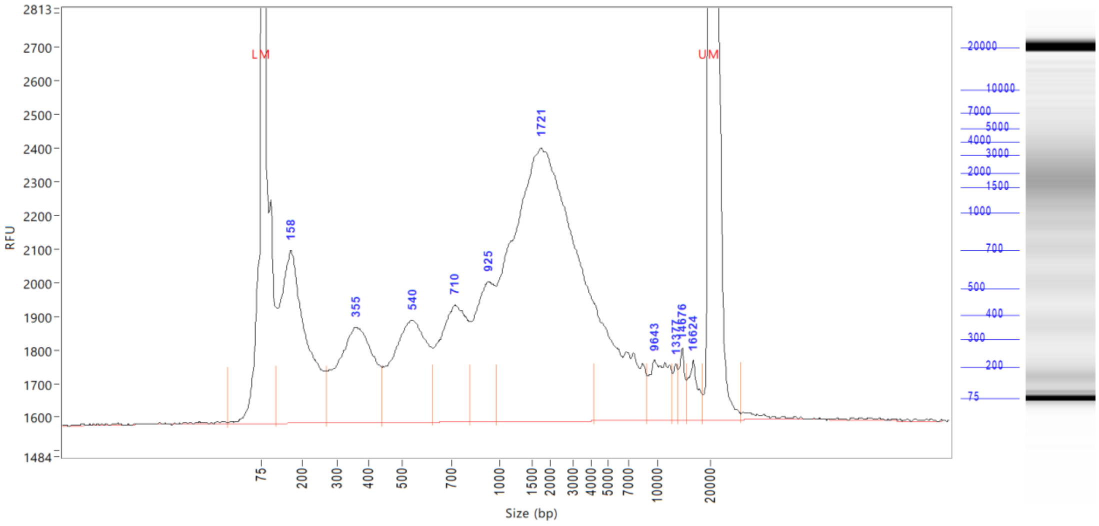

LM
UM
RFU
Size (bp)
158
355
540
710
925
1721
9643
16624

S5

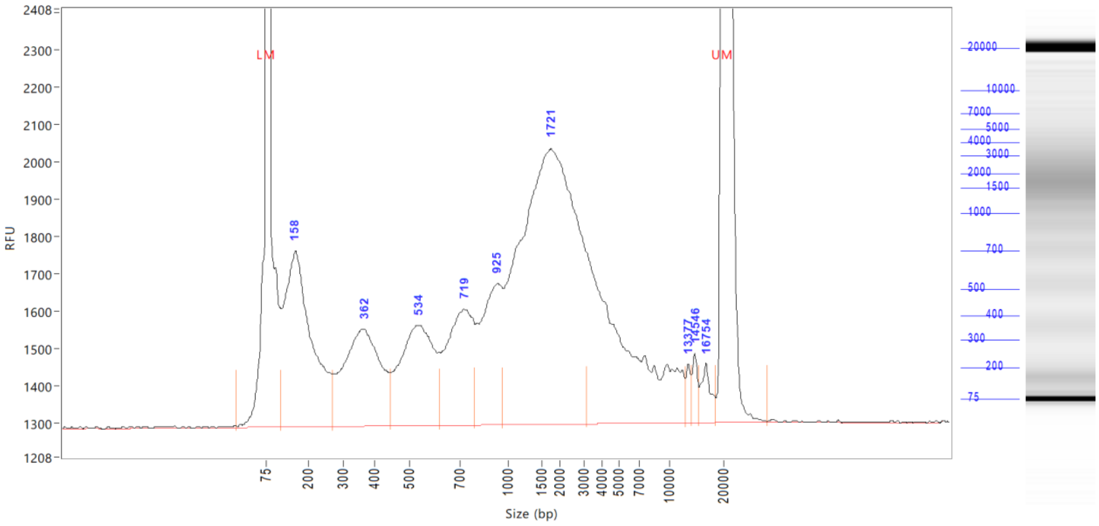

LM
UM
RFU
Size (bp)
158
362
534
719
925
1721
16754

S6

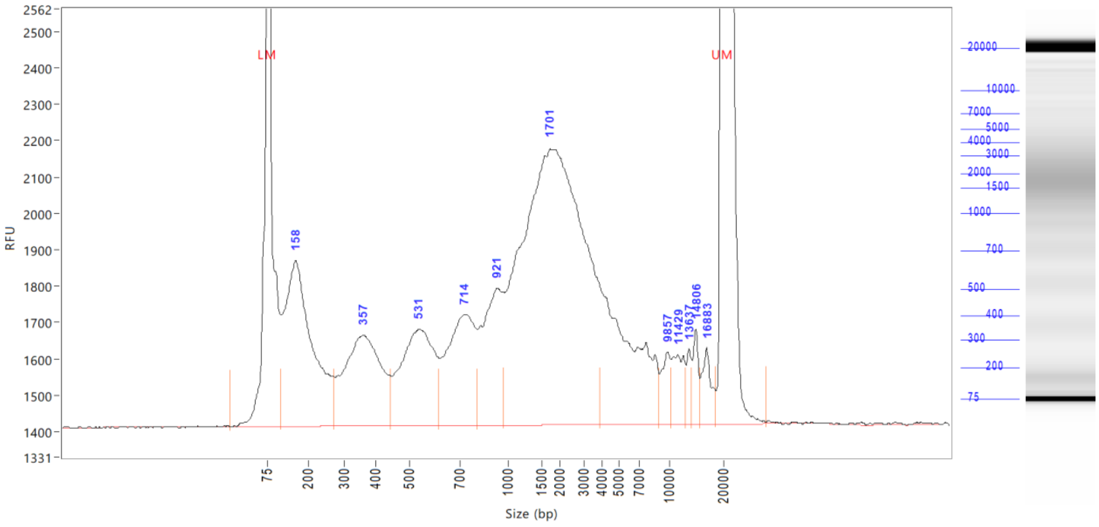

LM
UM
RFU
Size (bp)
158
357
531
714
921
1701
9857
11429
16883

S7

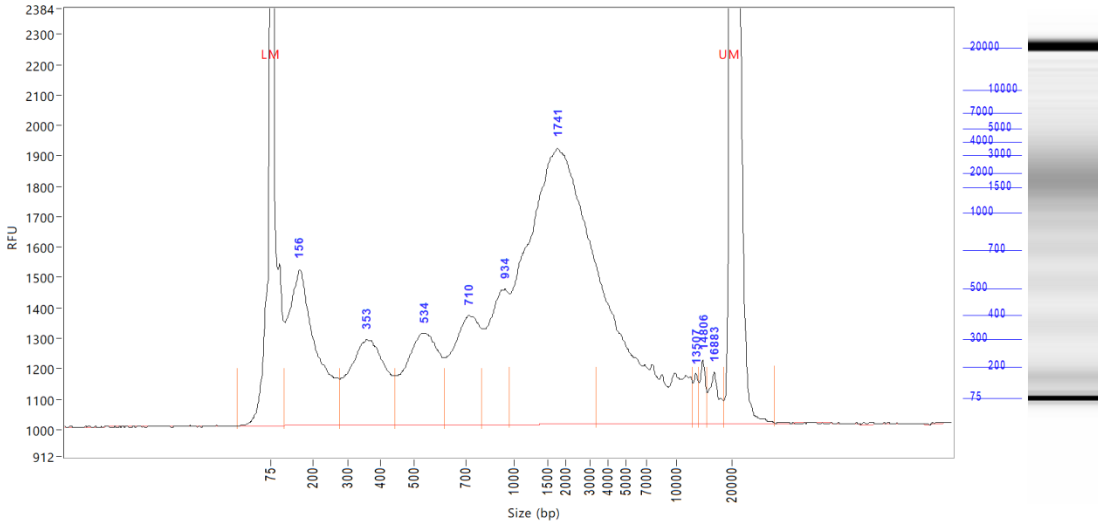

RFU
Size (bp)
LM
UM
156
353
534
710
934
1741
13507
14806
16883

S8

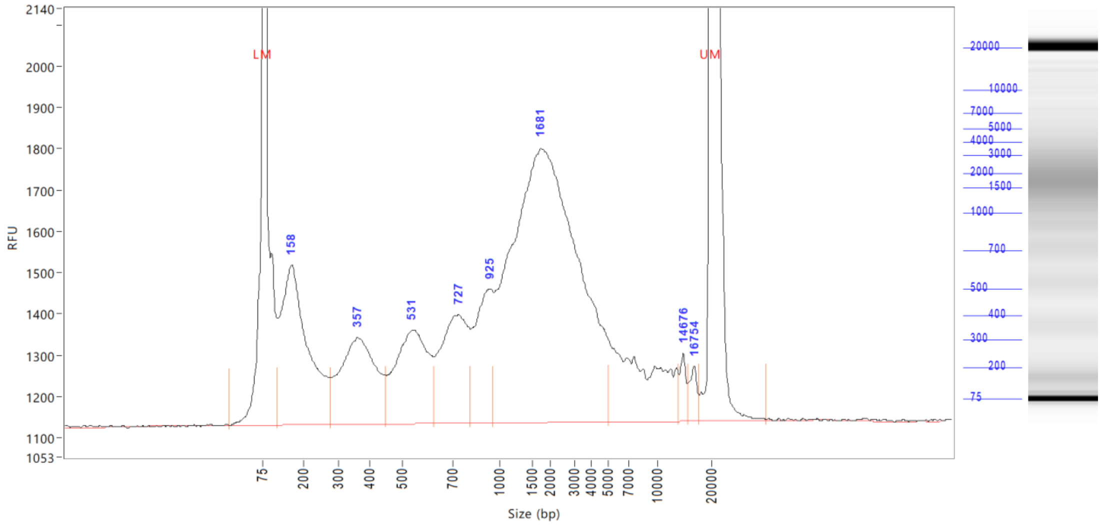

RFU
Size (bp)
LM
UM
158
357
531
727
925
1681
14676
16754

S9

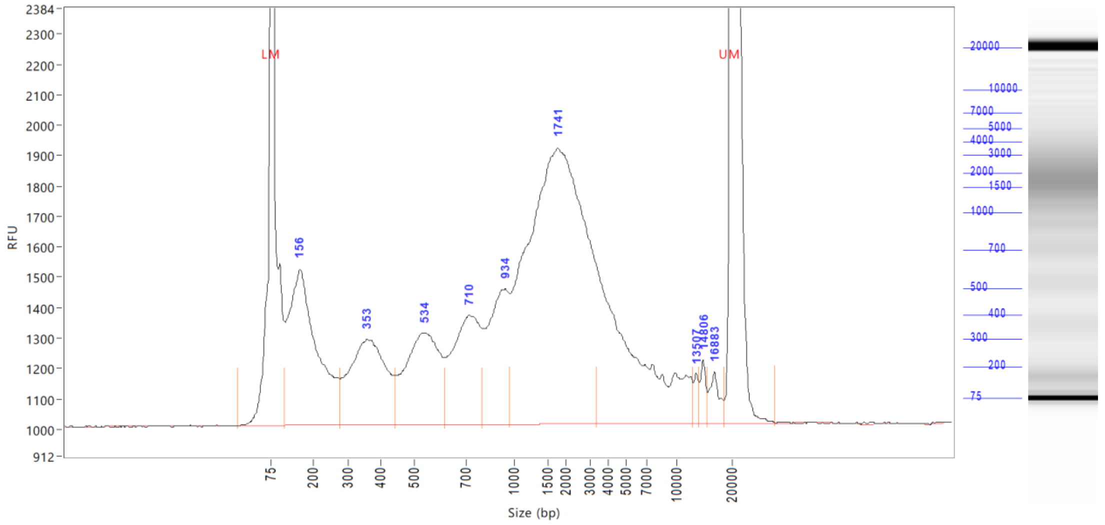

RFU
Size (bp)
LM
UM
156
353
534
710
934
1741
13507
14806
16883

S10

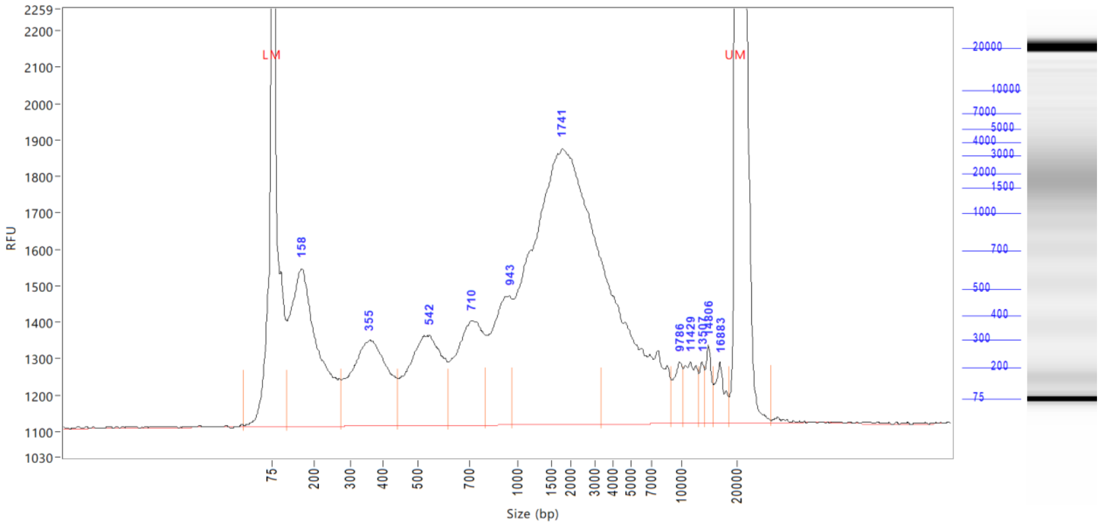

RFU
Size (bp)
LM
UM
158
355
542
710
943
1741
9786
11429
13507
14806
16883

S11

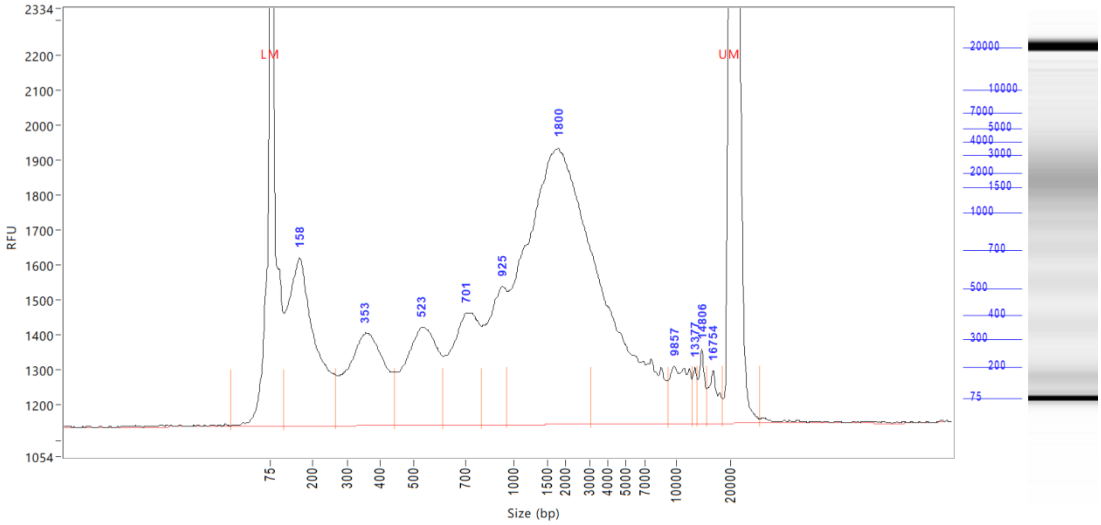

RFU
Size (bp)
LM
UM
158
353
523
701
925
1800
9857
13377
14806
16754

S12

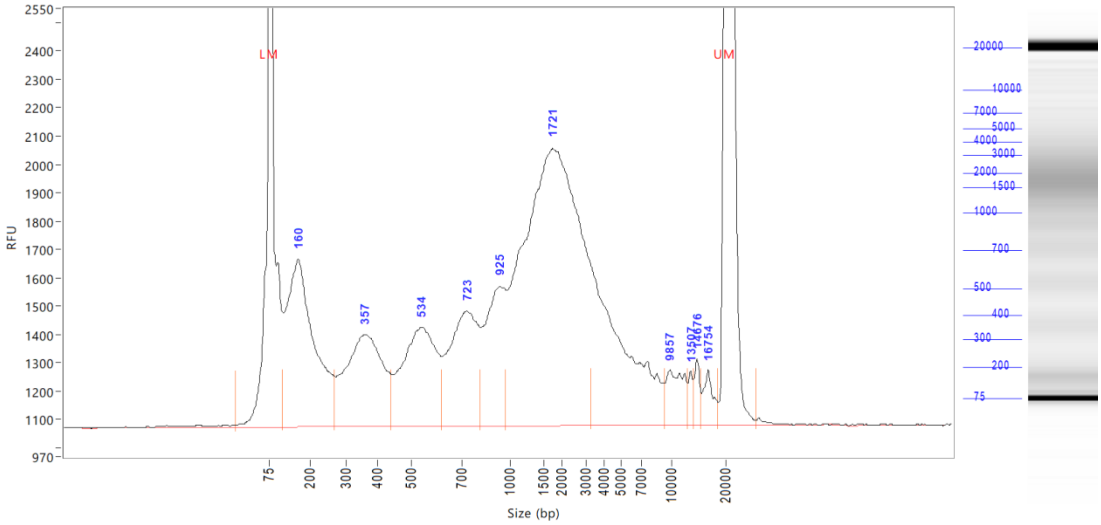

RFU
Size (bp)
LM
UM
160
357
534
723
925
1721
9857
13507
14676
16754

Supplementary figures representing the results of the **Fragment Analyzer DNF-915-33 (35-5000 bp)** assays. (S13) Ladder; (S14-S19) Biological replicate 1; and (S20-S24) Biological replicate 2.

S13

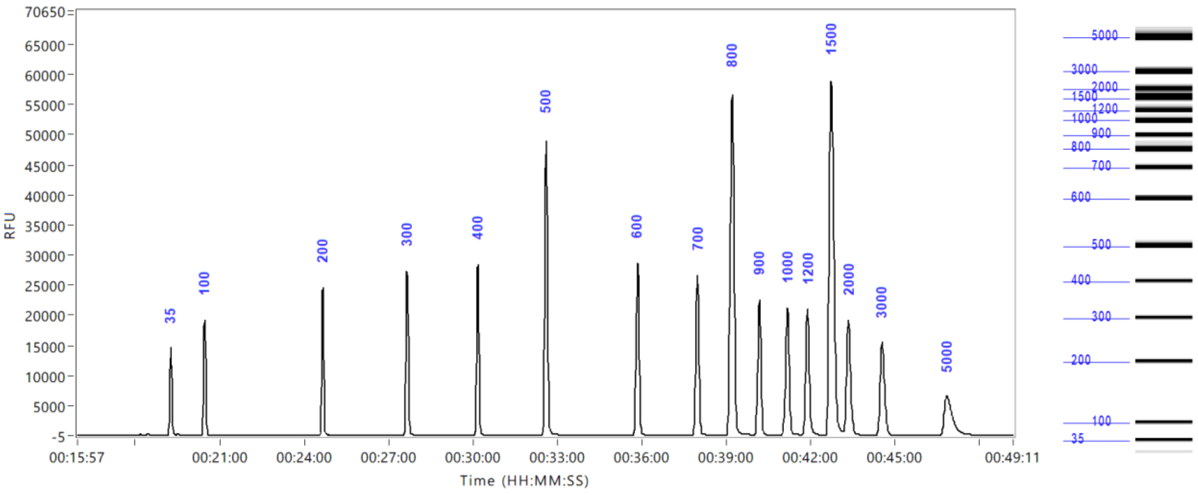

S14

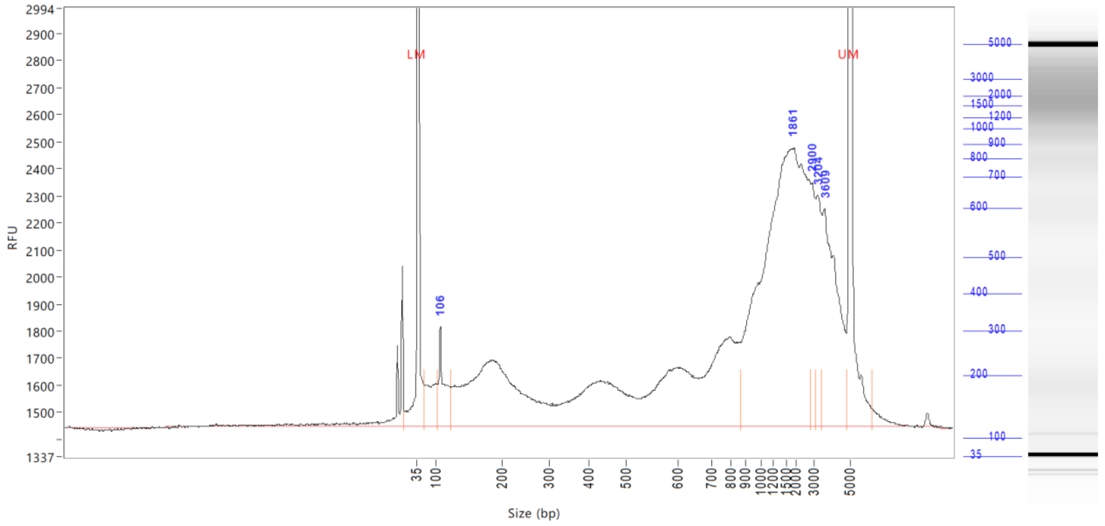

S15

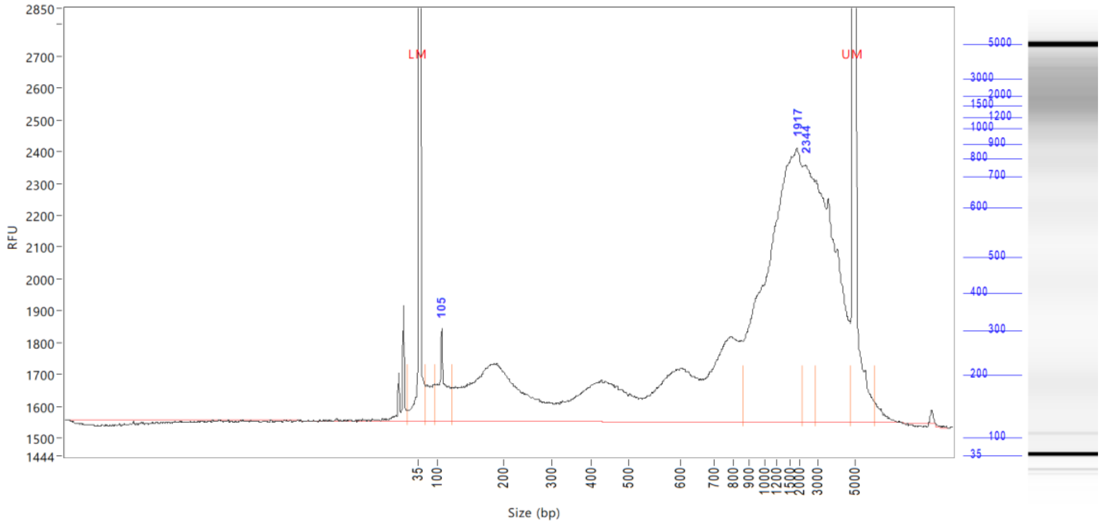

S16

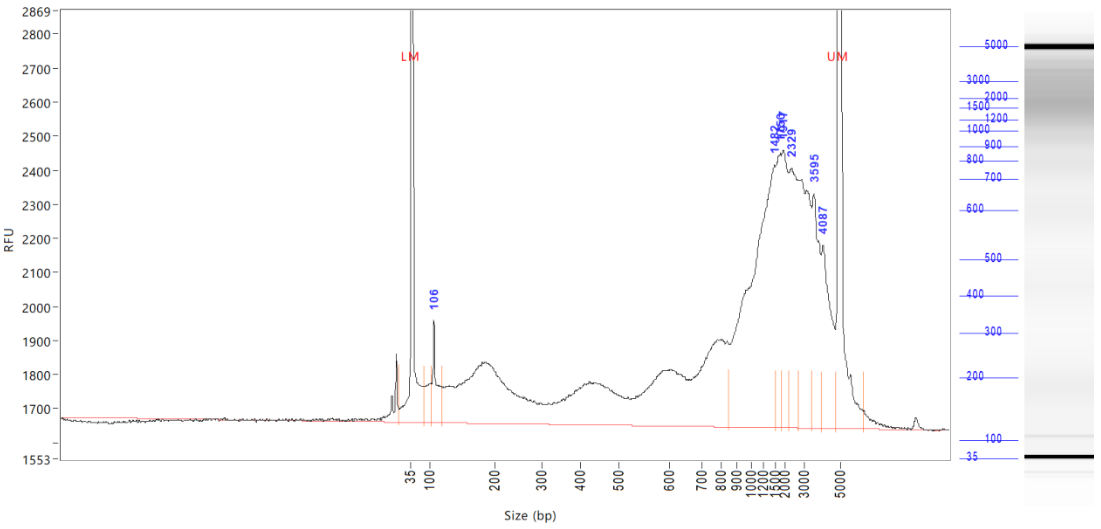

RFU
LM
UM
106
1482
2329
3595
4087
Size (bp)

S17

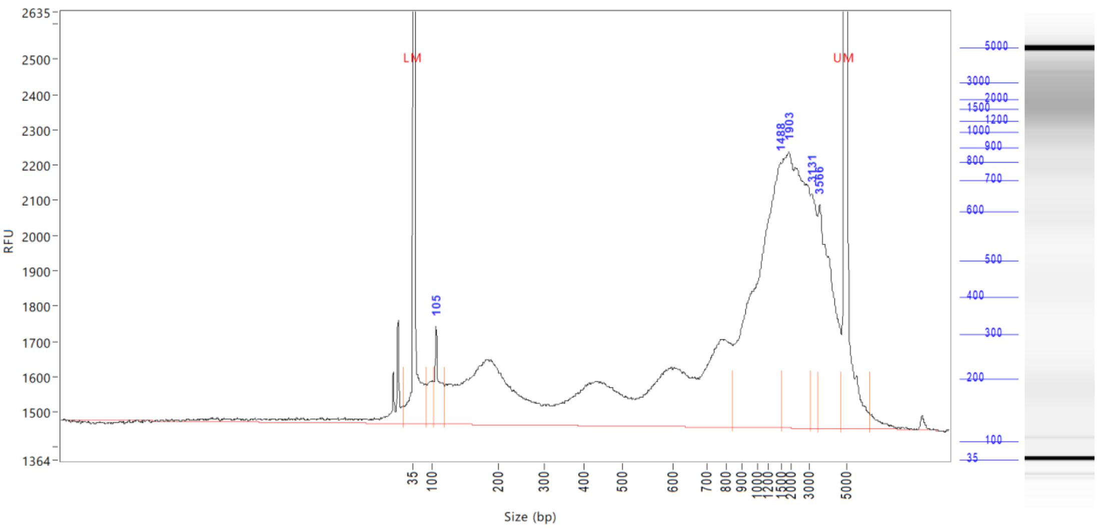

RFU
LM
UM
105
1488
1903
3131
3566
Size (bp)

S18

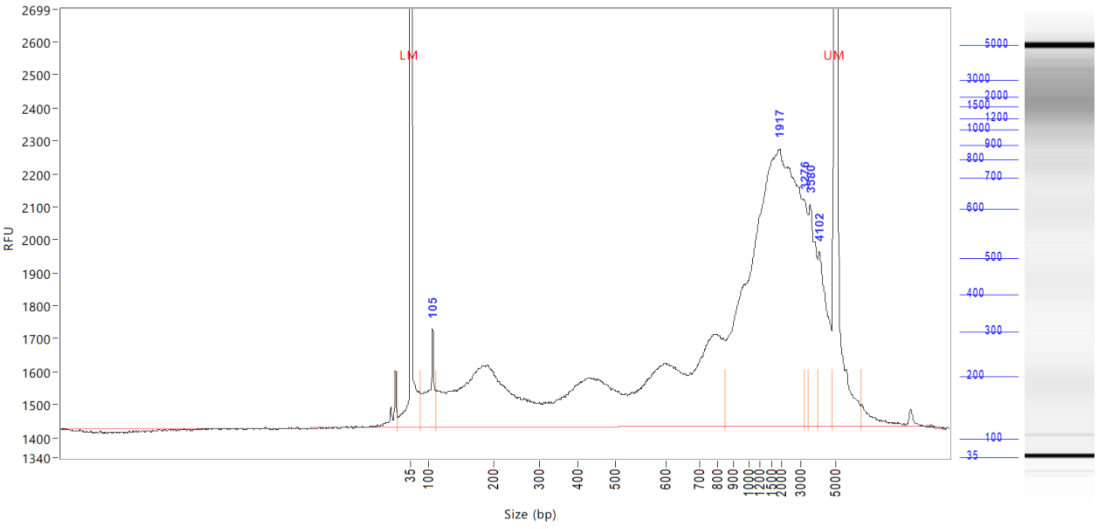

RFU
LM
UM
105
1917
3276
3580
4102
Size (bp)

S19

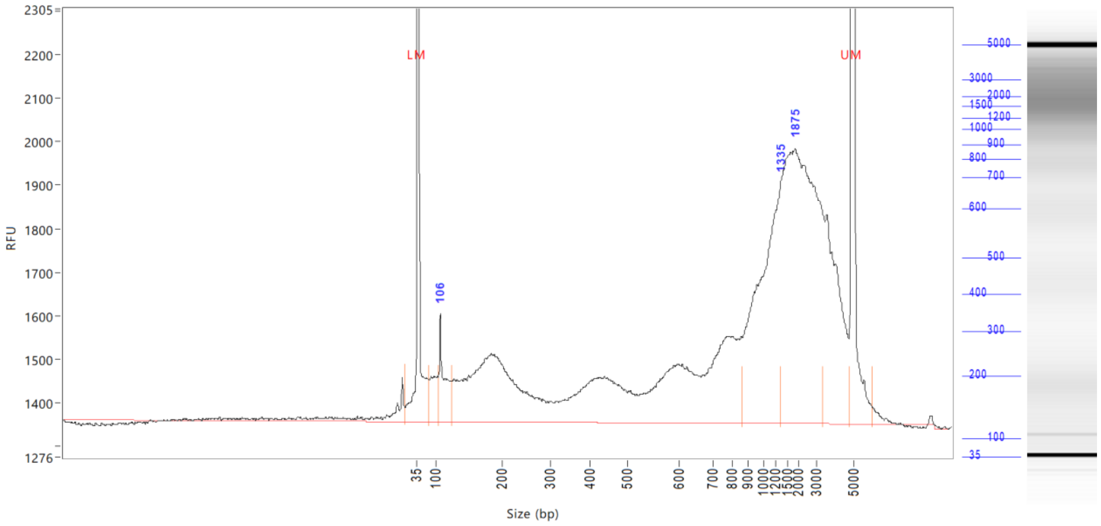

RFU
Size (bp)
LM
UM
106
1335
1875

S20

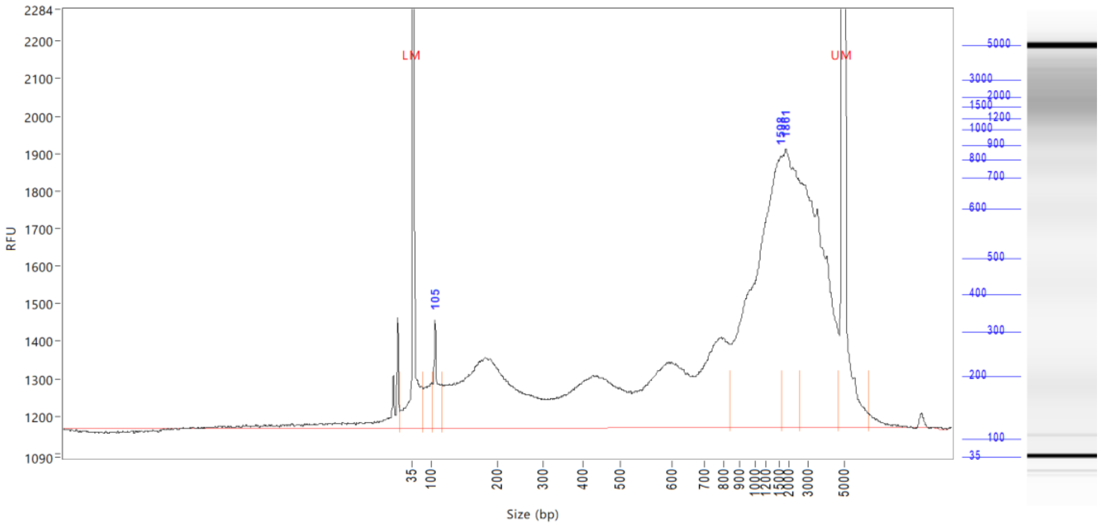

RFU
Size (bp)
LM
UM
105
1508
1881

S21

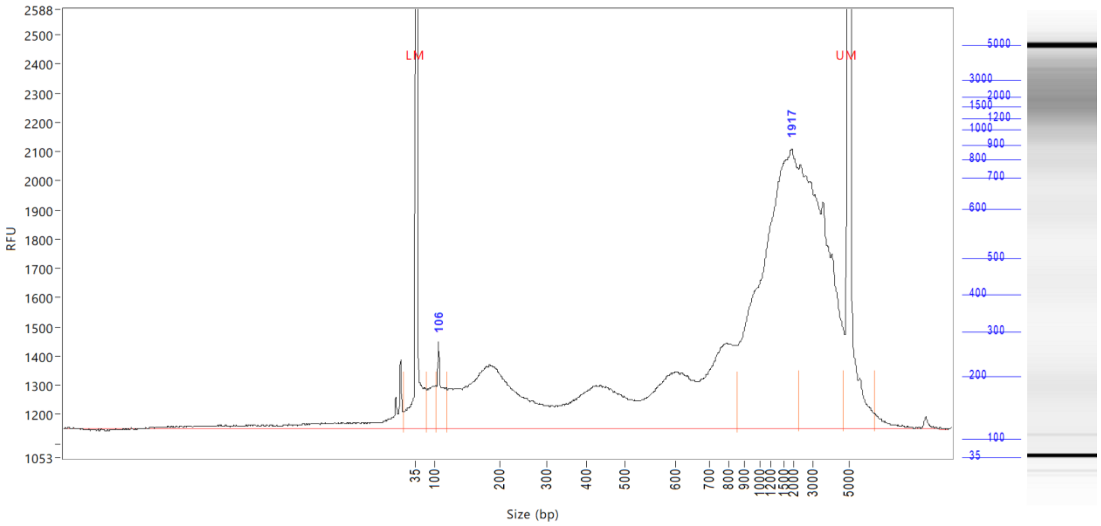

RFU
Size (bp)
LM
UM
106
1917

S22

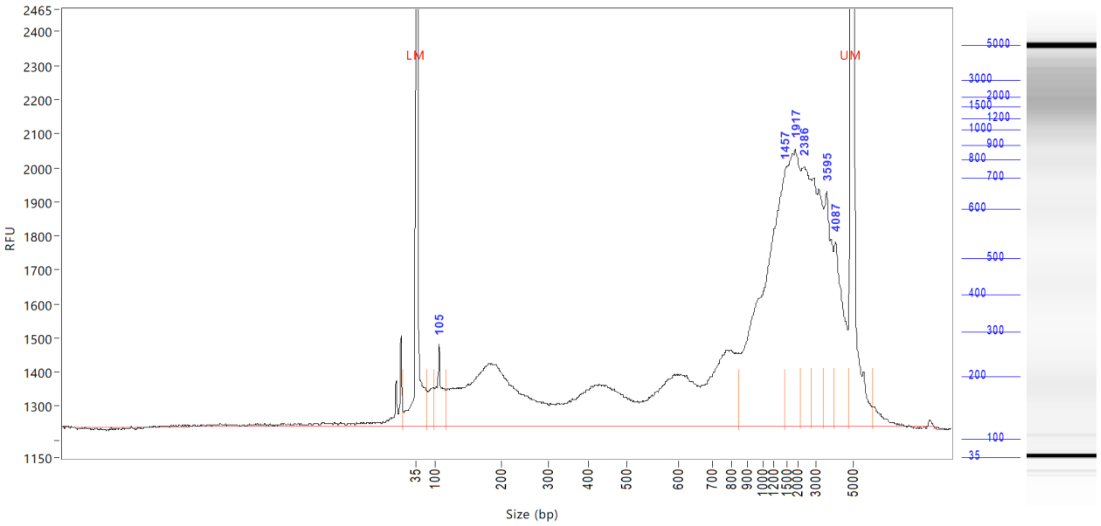
RFU
Size (bp)
LM
UM
105
1457
1917
2386
3595
4087

S23

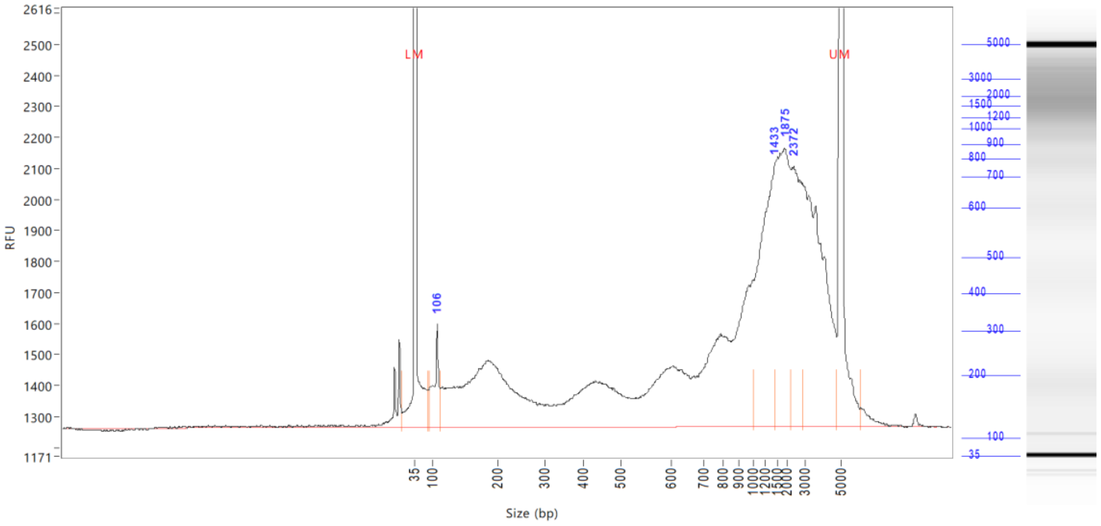
RFU
Size (bp)
LM
UM
106
1433
1875
2372

S24

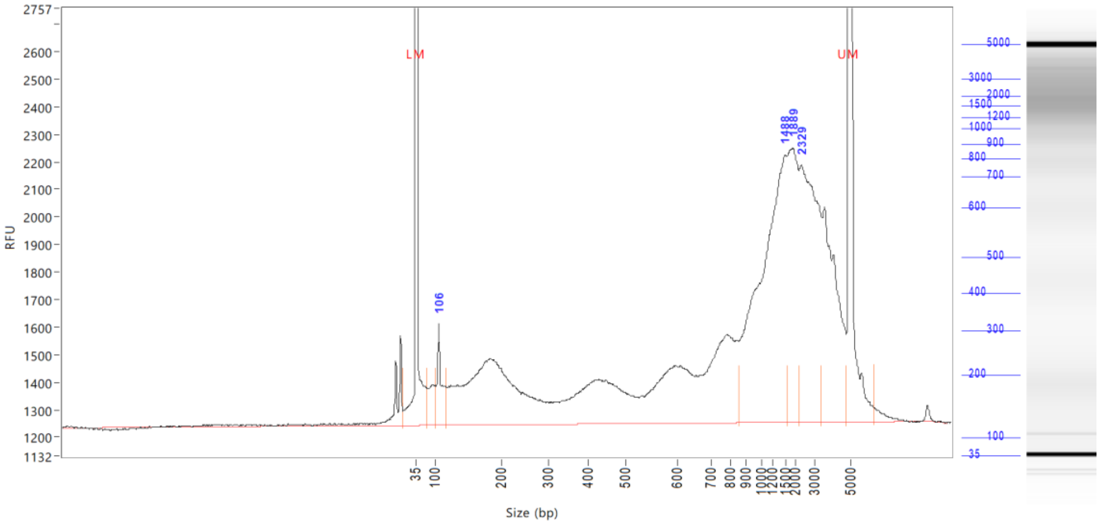
RFU
Size (bp)
LM
UM
106
1488
1889
2329

Supplementary figures representing the results of the **Bionalyzer DNA 1000 (25-1000 bp)** assay. (S25) Biological replicate 1; (S26) Biological replicate 2; and (S27) Biological replicate 3.

S25

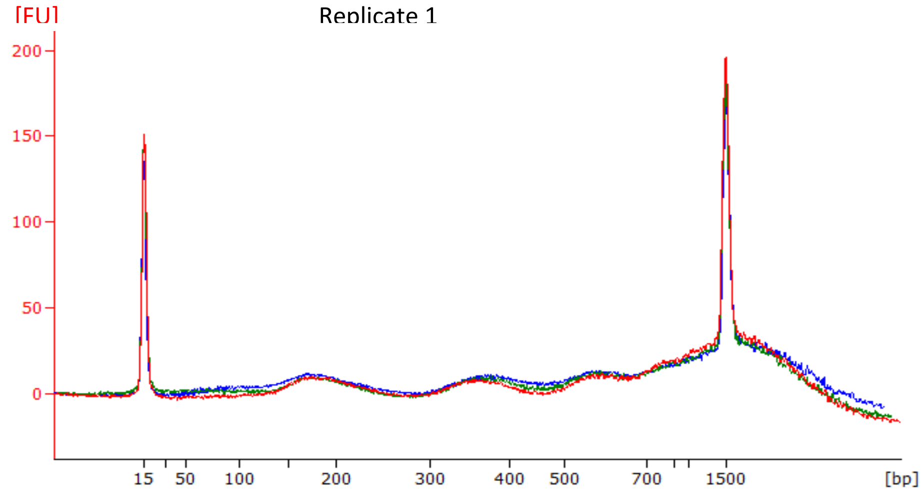

S26

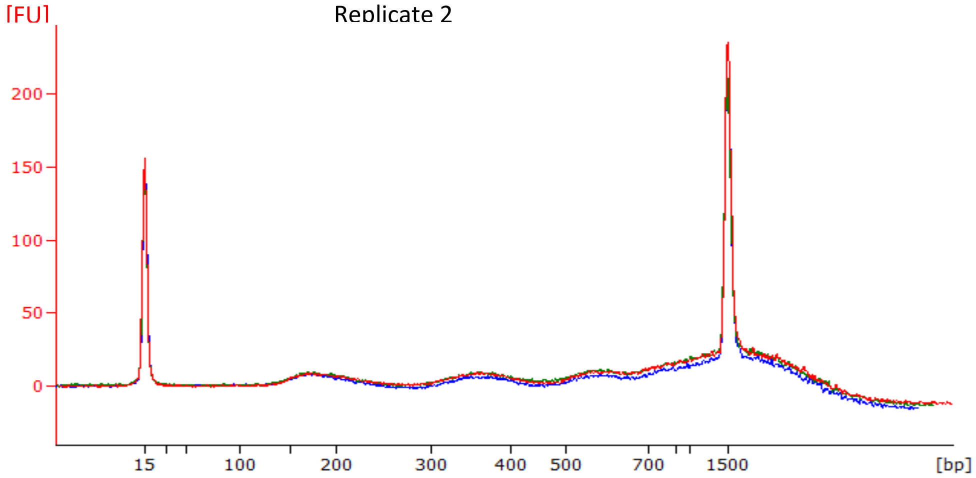

S27

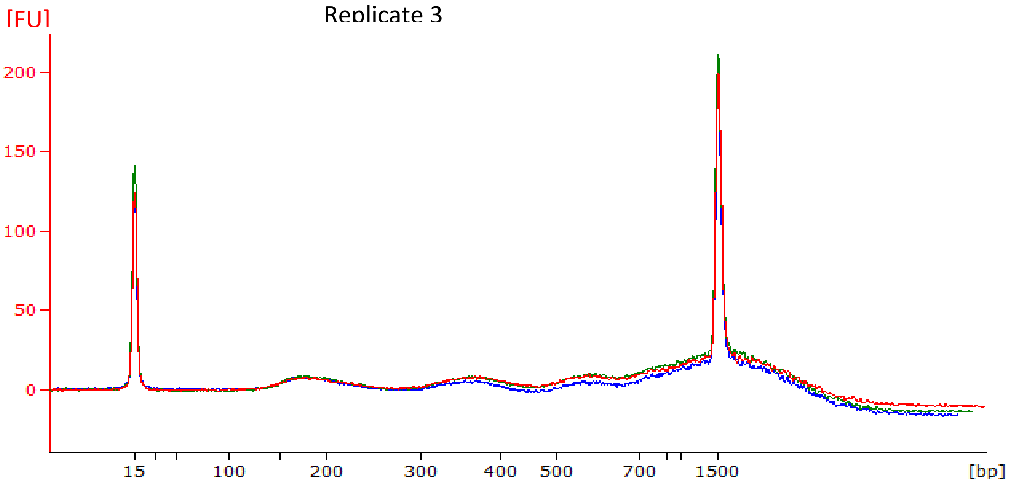

Supplementary figures representing the results of the **Bionalyzer High Sensitivity DNA Assay (50 – 7000 bp**. (S28) Biological replicate 1; and (S29) Biological replicate 2.

S28

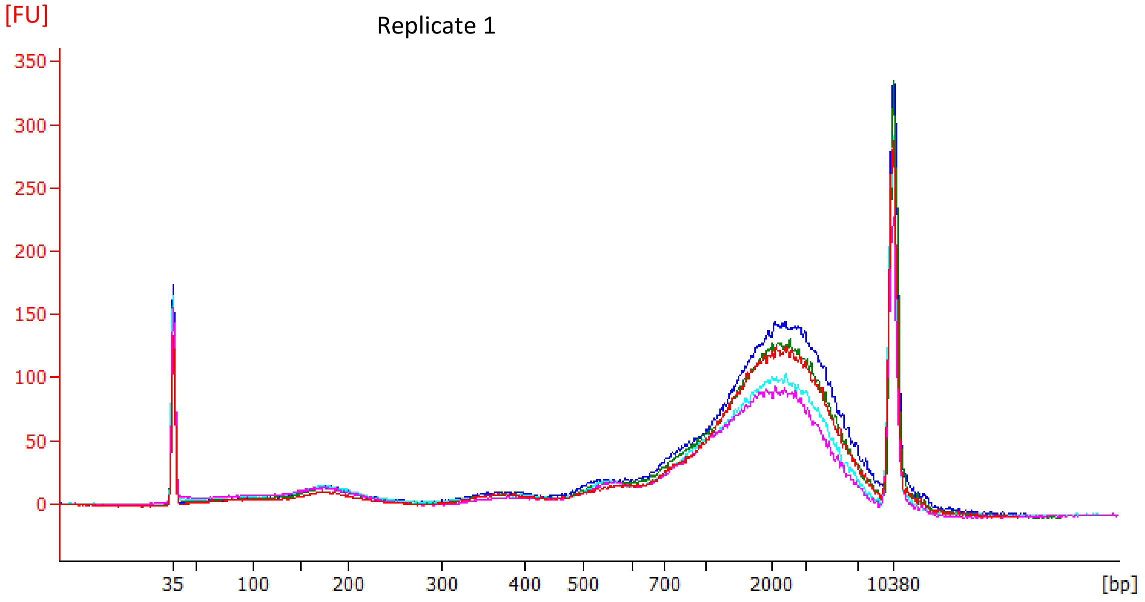

S29

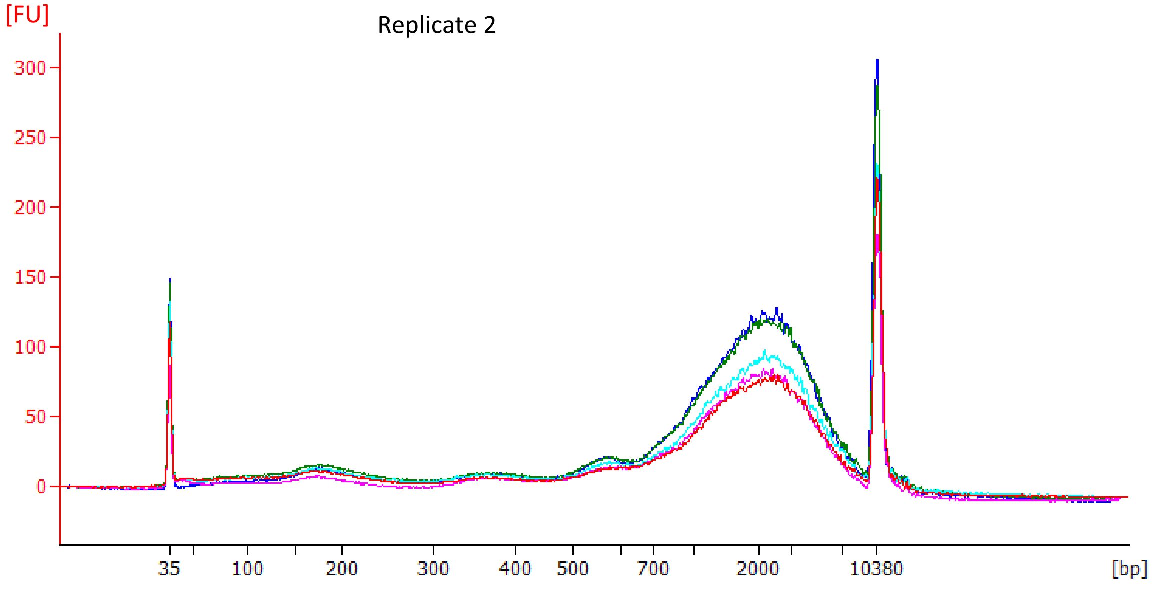

S30

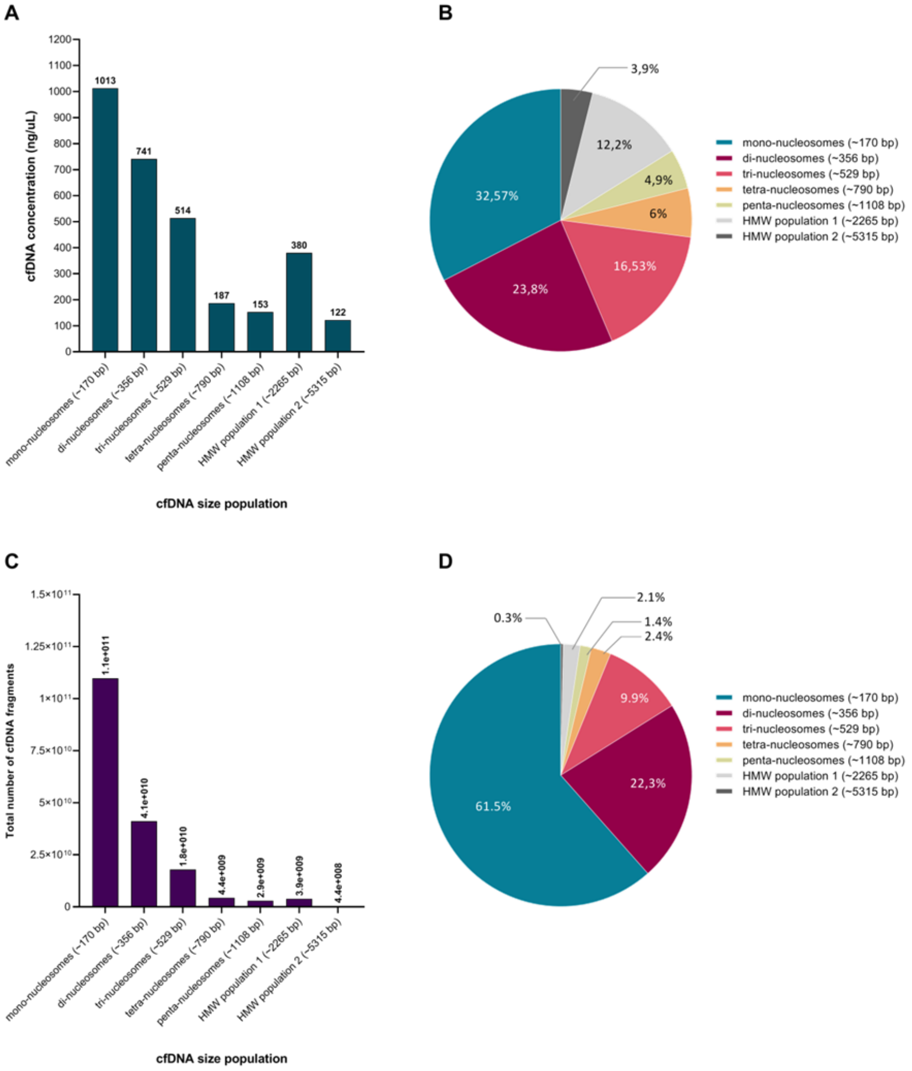

Supplementary Figure S30. Characterization of differently sized cell-free DNA (cfDNA) populations separated by agarose gel electrophoresis. Summary of the (**A**) average concentration, (**B**) relative contribution (%) in terms of concentration, (**C**) copy number (total number of cfDNA fragments) frequency distribution, and (**D**) relative contribution (%) in terms of copy number of the differently sized cfDNA populations present in the cell culture supernatant as determined by agarose gel electrophoresis coupled with the Agilent Bioanalyzer HS DNA assay. The total number of individual fragments that constitute each of the differently sized cfDNA populations (copy number) was determined as follows: (i) calculate the total number of base pairs that constitute each size population by dividing the average concentration of the population by the theoretical weight of 1 base pair (650 Da = 1.67 x 10-24 grams), and (ii) divide the total number of base pairs by the corresponding modal length of the population.
